# The genome of *Bacillus tequilensis* EA-CB0015 sheds light into its epiphytic lifestyle and potential as a biocontrol agent

**DOI:** 10.3389/fmicb.2023.1135487

**Published:** 2023-03-27

**Authors:** Tatiana Z. Cuellar-Gaviria, Camilo García-Botero, Kou-San Ju, Valeska Villegas-Escobar

**Affiliations:** ^1^CIBIOP Group, Department of Biological Sciences, Universidad EAFIT, Medellin, Colombia; ^2^Department of Microbiology, The Ohio State University, Columbus, OH, United States; ^3^Banana Research Center, Augura, Conjunto Residencial Los Almendros, Carepa, Colombia; ^4^Division of Medicinal Chemistry and Pharmacognosy, The Ohio State University, Columbus, OH, United States; ^5^Center for Applied Plant Sciences, The Ohio State University, Columbus, OH, United States; ^6^Infectious Diseases Institute, The Ohio State University, Columbus, OH, United States

**Keywords:** *Bacillus tequilensis*, comparative genomics, biocontrol, natural products, banana epiphyte

## Abstract

Different *Bacillus* species have successfully been used as biopesticides against a broad range of plant pathogens. Among these, *Bacillus tequilensis* EA-CB0015 has shown to efficiently control Black sigatoka disease in banana plants, presumably by mechanisms of adaptation that involve modifying the phyllosphere environment. Here, we report the complete genome of strain EA-CB0015, its precise taxonomic identity, and determined key genetic features that may contribute to its effective biocontrol of plant pathogens. We found that *B. tequilensis* EA-CB0015 harbors a singular 4 Mb circular chromosome, with 3,951 protein-coding sequences. Multi-locus sequence analysis (MLSA) and average nucleotide identity (ANI) analysis classified strain EA-CB0015 as *B. tequilensis*. Encoded within its genome are biosynthetic gene clusters (BGCs) for surfactin, iturin, plipastatin, bacillibactin, bacilysin, subtilosin A, sporulation killing factor, and other natural products that may facilitate inter-microbial warfare. Genes for indole-acetic acid (IAA) synthesis, the use of diverse carbon sources, and a multicellular lifestyle involving motility, biofilm formation, quorum sensing, competence, and sporulation suggest EA-CB0015 is adept at colonizing plant surfaces. Defensive mechanisms to survive invading viral infections and preserve genome integrity include putative type I and type II restriction modification (RM) and toxin/antitoxin (TA) systems. The presence of bacteriophage sequences, genomic islands, transposable elements, virulence factors, and antibiotic resistance genes indicate prior occurrences of genetic exchange. Altogether, the genome of EA-CB0015 supports its function as a biocontrol agent against phytopathogens and suggest it has adapted to thrive within phyllosphere environments.

## Introduction

Endospore forming bacteria of the genus *Bacillus* are ubiquitous within terrestrial and aquatic environments (Nicholson, 2002). Industrially important species, including *B. subtilis*, *B. amyloliquefaciens*, *B. velezensis, B. licheniformis*, and *B. pumilus,* form a phylogenetically coherent group known as the *B. subtilis* species complex (Fritze, 2004). In addition to being an important source of industrial enzymes, vitamins, and cofactors (Harwood et al., 2018), many of these species are used as the active ingredient in several commercial biopesticides and biofertilizers (Velivelli et al., 2014; Ngalimat et al., 2021). Well known strains including *B. amyloliquefaciens* FZB42 (reclassified as *B. velezensis*; Chen et al., 2009), *B. subtilis* QST713 (recently reclassified as *B. velezensis*; Pandin et al., 2018), *B. amyloliquefaciens* GB03 (Choi et al., 2014), and *B. subtilis* MBI600 (Samaras et al., 2021) are used to control soil and foliar bacterial and fungal diseases. These strains provide a natural alternative to synthetic agrochemicals that have detrimental effects to human health and the environment.

The utility of *Bacilli* as biocontrol and bioaugmentation agents derives from traits inherent to their natural lifestyle as microbial endo- and epiphytes. These bacteria localize to plants *via* directed movement to phytochemicals (e.g., chemotaxis to organic acids such as malic and fumaric acid; Tsai et al., 2020; Ma et al., 2021) and then use catabolic pathways to consume these compounds as carbon, nitrogen, and energy sources for growth. Colonization of plant surfaces and tissues occurs through the establishment of biofilms (Rudrappa et al., 2008). Equally significant are the natural products *Bacilli* produce and their effects on modulating plant development and the composition of resident microbiomes. *Bacilli* produce phytohormones including auxins (indole acetic acid, IAA), cytokinins, and gibberellins that regulate plant growth and differentiation. These compounds are important modulators of many plant processes ranging from abiotic stress response to flowering, fruit development, and seed germination (Poveda and González-Andrés, 2021). Other antimicrobial natural products such as cyclic lipopeptides (surfactin, iturin, and fengycin/plipastatin), siderophores (bacillibactin), bacteriocins (sublancin, subtilosin; Stein, 2005) may provide ecological advantages to the *Bacilli* by suppressing fungal and bacterial competitors. The importance of these compounds is further underscored by the abundance and conservation of natural product biosynthetic gene clusters (BGCs) within the genomes of plant-associated *Bacilli*. Indeed, *B. velezensis* QST713 harbors 15 natural product BGCs (Pandin et al., 2018), while *B. amyloliquefaciens* GB03 and *B. subtilis* MBI600 harbor 8 and 7 BGCs, respectively (Choi et al., 2014; Samaras et al., 2021).

While most species in the *B. subtilis* complex have been well characterized, significantly less is understood about the physiology and ecology of *B. tequilensis*. First described in 2006, emergent interest in *B. tequilensis* has been driven by its natural ability to suppress diverse fungal pathogens of commercial grains, vegetables, fruits, and ornamentals (Tam et al., 2020; Xu et al., 2021; Zhou et al., 2021; Kwon et al., 2022). Among *B. tequilensis*, one of the best characterized strains is EA-CB0015. Originally isolated in Uraba, Colombia, EA-CB0015 was discovered as a natural antagonist of *Pseudocercospora fijiensis* (Ceballos et al., 2012), the causative agent of black Sigatoka. This agriculturally devastating disease of banana plants causes necrotic streaks on the leaves, reduces photosynthetic capacity, and promotes premature ripening of the fruit. The resulting loses can be greater than 50% if left untreated (Noar et al., 2022). Mitigation of black Sigatoka represents a significant burden to Colombian producers, with a cost $65 million per year due to weekly application of fungicides (S. Zapata, personal communication). EA-CB0015 suppresses black Sigatoka disease through the colonization of banana leaves and the production of antifungal lipopeptides (iturins, plipastatins, and surfactins; Villegas-Escobar et al., 2013; Mosquera et al., 2014; Cuellar-Gaviria et al., 2021). In addition to bananas, the strain also reduced the severity of anthracnose (*Colletotrichum* spp.) in tamarillo fruits and gray mold (*Botrytis cinerea*) in chrysanthemum flowers (Gutierrez-Monsalve et al., 2015; Arroyave-Toro et al., 2017). Although *B. tequilensis* has yet to be commercialized, these studies collectively demonstrate the significant potential of this species for the control and prevention of agricultural diseases.

Here we sequenced the complete genome of strain EA-CB0015 to understand the genetic features integral to its success as a microbial epiphyte and biocontrol agent. Using different molecular taxonomy approaches, we show that EA-CB0015 is classified as *B. tequilensis*. Our analysis and comparisons of its genome with other plant associated *Bacilli* identified mechanisms for plant colonization and the inhibition of phytopathogens. These include genes for sporulation, biofilm formation, and the ability to metabolize diverse nutrient sources. Distinct mechanisms of protection against abiotic and biotic factors were present, including genes that direct biosynthesis of an arsenal of natural products. Lastly, features related to horizontal gene transfer including the presence of genomic islands (GEIs), insertion sequences (IS), toxin antitoxin (TA) systems, restriction modification (RM) system, and prophages are described.

## Materials and methods

### Genome sequencing and assembly

*Bacillus tequilensis* EA-CB0015 was deposited in ATCC as PTA-123533. The strain was revived from glycerol stocks onto half strength tryptic soy agar [20 g/L of TSA (Oxoid) and 9 g/l of Bacto agar (BD)] and incubated at 37°C for 24 h. A single colony was inoculated into a 100 mL Erlenmeyer containing 20 mL of tryptic soy broth (Oxoid) and grown for 8 h in a shaking incubator (250 rpm, 37°C). Genomic DNA was extracted using the UltraClean Microbial DNA Isolation kit (MoBio) for Illumina sequencing and DNeasy Blood & Tissue kit (Qiagen) for Single Molecule Real-Time (SMRT) sequencing. Samples were sequenced on an Illumina HiSeq 2000 (100 bp paired end reads) and PacBio RS II (Macrogen, Inc). Contigs less than 100 bp were discarded. Unicycler v0.4.8.0 was used for hybrid assembly using the normal bridging mode (Wick et al., 2017).

### General annotation

The assembled genome was annotated using the NCBI Prokaryotic Genome Annotation Pipeline (PGAP) v6.0 (Haft et al., 2018) and deposited under accession no. NZ_CP048852. *Skew*DB was used to predict replication origins and termini (Hubert, 2022). Genome figures were created using the CGView Server (Grant and Stothard, 2008). Predicted proteins were classified into clusters of orthologous groups (COGs) using WebMGA (Wu et al., 2011), and analyzed in the Kyoto Encyclopedia of Genes and Genomes database (KEGG; entry T07089) to assign roles in metabolic pathways (Kanehisa et al., 2016).

### Multi-locus sequence analysis

All strains analyzed in this study are listed in Supplementary Table S1. Reference strains included *B. subtilis* species complex (*B. pumilus, B. subtilis*, *B. tequilensis*, *B. mojavensis*, *B. atrophaeus*, *B. velezensis*, and *B. amyloliquefaciens, B. licheniformis*), the *B. cereus* species complex (*B. cereus, B. thuringiensis, B. anthracis*, *B. mycoides*), and *B. coagulans*. *Clostridium kluyveri* DSM 555^T^ served as the outgroup.

Genomes were downloaded from public sequence repositories and annotated using Prokka v1.14.6 (Seemann, 2014). Genes encoding *groEL* (chaperonin, large subunit), *gyrA* (DNA gyrase, subunit A), *rpoB* (RNA polymerase, beta subunit)*, polC* (DNA polymerase III, alpha subunit), and *purH* (phosphoribosylaminoinidazole carboxamide formyltransferase) were retrieved from each strain, aligned using MAFFT v7.4 (Katoh, 2002), manually trimmed, and concatenated head to tail using AMAS v0.98 (Borowiec, 2016). The matrix (12,483 positions; 1,647 from *groEL,* 2,517 from *gyrA,* 3,030 from *polC,* 1,542 from *purH*, and 3,747 from *rpoB*) was analyzed using ModelTest-NG v0.1.6 (Darriba et al., 2020) to determine the best substitution model for each partition. Phylogenetic trees were constructed using maximum-likelihood (ML) and Bayesian inference (BI) methods. The ML phylogeny using the GTR + I + G4 model was best for *groEL, gyrA, purH* and *rpoB,* and GTR + G4 for *polC,* with bootstrapping (1,000) using IQ-TREE v1.6.9 (Nguyen et al., 2015). The BI phylogeny was calculated using Markov Chain Monte Carlo (MCMC) analysis and the GTR + I + G4 model using Mr. Bayes v.3.2 (Ronquist et al., 2012). Two independent runs were performed for 10 million generations (1 sampling every 1,000 generations). Effective sample size, convergency, and stationarity values were evaluated using Tracer v1.7.1 (Rambaut et al., 2018). The final phylogenetic tree was generated using DendroPy v4.4.0 (Sukumaran and Holder, 2010) and visualized using iToL v5.0 (Letunic and Bork, 2019).

### Growth assays

The ability of strain EA-CB0015 to utilize different carbon sources was performed using a BioLog GEN III Microplate (Biolog Inc.). EA-CB0015 was grown on Universal Growth Agar (BUG; Biolog Inc.) and incubated at 30°C for 24 h. Isolated colonies were resuspended in the “Inoculation Fluid-A” (IF-A; Biolog Inc.) following manufacturer’s instructions and this suspension was inoculated into the Gen III microplate. After 24 h incubation at 30°C, the microplate was analyzed qualitatively for color development using a MicroStation™ 2 Reader (Biolog Inc.), measuring OD at 595 and 750 nm.

### Sporulation, motility, and biofilm formation assays

Colonies grown on TSA plates were stained for endospores using Schaeffer-Fulton method (Gerhardt et al., 1994). Micrographs were recorded using an Axiostar Plus microscope (Carl Zeiss) at 100X equipped with an AxioCam ICc3 (Carl Zeiss) and ZEN 2.3 lite software (Carl Zeiss). Biofilm formation was examined by inoculating a diluted culture of EA-CB0015 into 12-well plates with LBGM media. These were incubated for 48 h at 30°C (statically) and photographed (7.1 MP digital camera, Kodak EasyShare P712). Motility was assayed by inoculating 3 μl EA-CB0015 grown on tryptic soy broth TSB (Oxoid; OD 1.0, 600 nm) onto the center of 10% LB plates solidified with either 0.7% (swarming) or 0.3% agar (swimming), and incubated at 30°C for 24 h (Kearns and Losick, 2003; Ghelardi et al., 2012). Flagella were visualized using Ryu stain (Heimbrook et al., 1989).

### Analysis of natural product biosynthetic gene clusters

Genomes were analyzed using antiSMASH v5.1.2 (Blin et al., 2019). BGCs with similarity scores > 70% were reported for all 58 *Bacillus* strains. Regions encoding with similarity scores less than <70% were further analyzed for strain EA-CB0015 using BLAST (Johnson et al., 2008) and HMMER v3.3.1 (Eddy, 2011). BGCs for natural products known to be produced by *Bacilli* but not detected by antiSMASH were manually identified by BLAST.

### Analysis of virulence factors, antibiotic resistance, and genetic exchange

Putative phage and phage-like regions were identified using PHASTER (Zhou et al., 2011; Arndt et al., 2016). Data ordering, sub-setting and reshaping was performed using Tidyverse v1.3.0 (Wickham et al., 2019) and an in-house script uploaded on GitHub^1^ (García-Botero et al., 2021). GEIs were identified using IslandViewer 4 (Bertelli et al., 2017) and ISs identified using ISfinder (e-value cutoff of 1e-07; Siguier et al., 2006). Integrative or conjugative elements (ICEBs1) were predicted with ICEBerg 2.0 (Liu M. et al., 2019). Antimicrobial resistance genes were identified using ResFinder-4.0 (70% identity threshold; minimum length of 70%; Bortolaia et al., 2020), and putative virulence factors from the Virulence Factors of Pathogenic Database (VFDB; Liu B. et al., 2019). RM systems were retrieved from REBASE (Roberts et al., 2015). Cluster BLAST analysis of identified RM systems were performed using Cblaster v1.3.0 (Gilchrist et al., 2021). Potential TA systems identified using TAfinder (Xie et al., 2018) and manually BLAST searches. Finally, CRISPRCasFinder (Couvin et al., 2018) was used to identify CRISPRs and Cas genes.

### Comparative genomics

Synteny was analyzed using Easyfig v2.2.2 (Sullivan et al., 2011). ANI values (Richter and Rosselló-Móra, 2009) were calculated using GTDB-Tk v0.3.2 (Chaumeil et al., 2019). Comparisons between *B. subtilis* 168, *B. tequilensis* ATCC BAA 819^T^ and *B. tequilensis* EA-CB0015 were performed using proteome and protein family functions in PATRIC (Davis et al., 2020). Presence or absence of predicted genes and proteins in EA-CB0015 were compared to *B. subtilis* 168 (NBCI Reference Sequence: NC_000964.3) using SubtiWiki (Zhu and Stülke, 2018). E-values < 1e-5 and sequence identities > 70% were used as cutoffs.

## Results

### The genome of EA-CB0015

**Figure 1:**
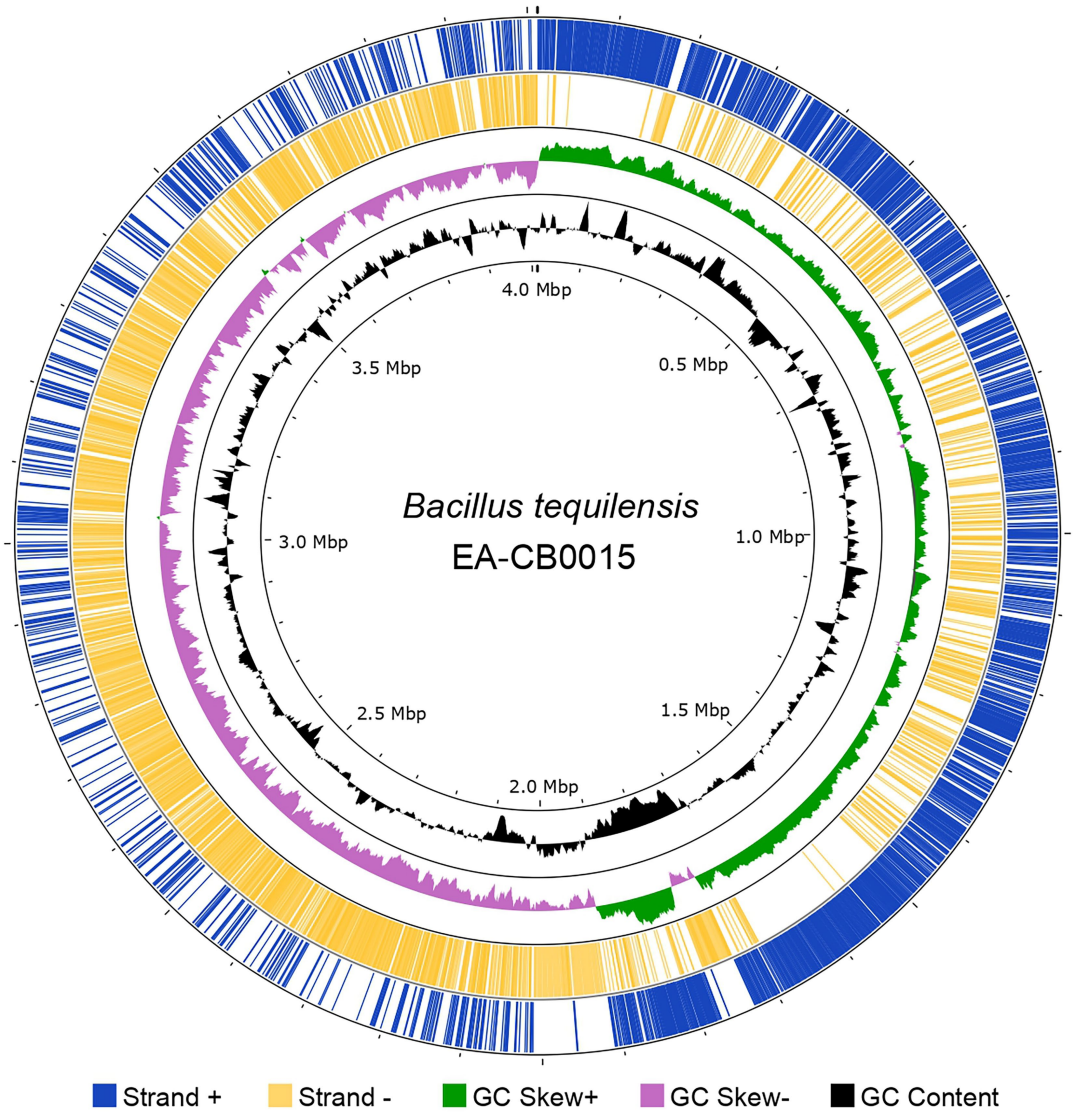
The genome of *Bacillus tequilensis* EA-CB0015. GenBank features (CDS) in the forward strand (blue) and reverse strand (yellow); GC skew (green and purple) and GC content (black). Image created with Proksee.

Hybrid assembly of Illumina and SMRT sequencing reads produced a single circularized chromosome of 4,012,371 bp with an average GC content of 43.7%. In total, 4,112 CDS (3,951 genes, 161 pseudogenes), 10 copies of rRNAs (5S, 16S, and 23S), 86 tRNAs, and 5 ncRNAs were present. The forward strand encoded 2,134 genes and the reverse strand 1,978 genes (Figure 1). GC-skew suggests single symmetrical, bi-directional replication of the genome (Supplementary Figure S1). COG analysis assigned these into 5,163 protein families, most of which belonged to general or unknown function (categories R and S). Besides these, the largest groups of proteins are dedicated for amino acid transport and metabolism (E), energy production and conversion (C), translation, ribosomal structure, and biogenesis (J), DNA replication, recombination, and repair (L), and transcription (K; Supplementary Figure S2).

### Molecular taxonomy classifies EA-CB0015 as *Bacillus tequilensis*

**Figure 2:**
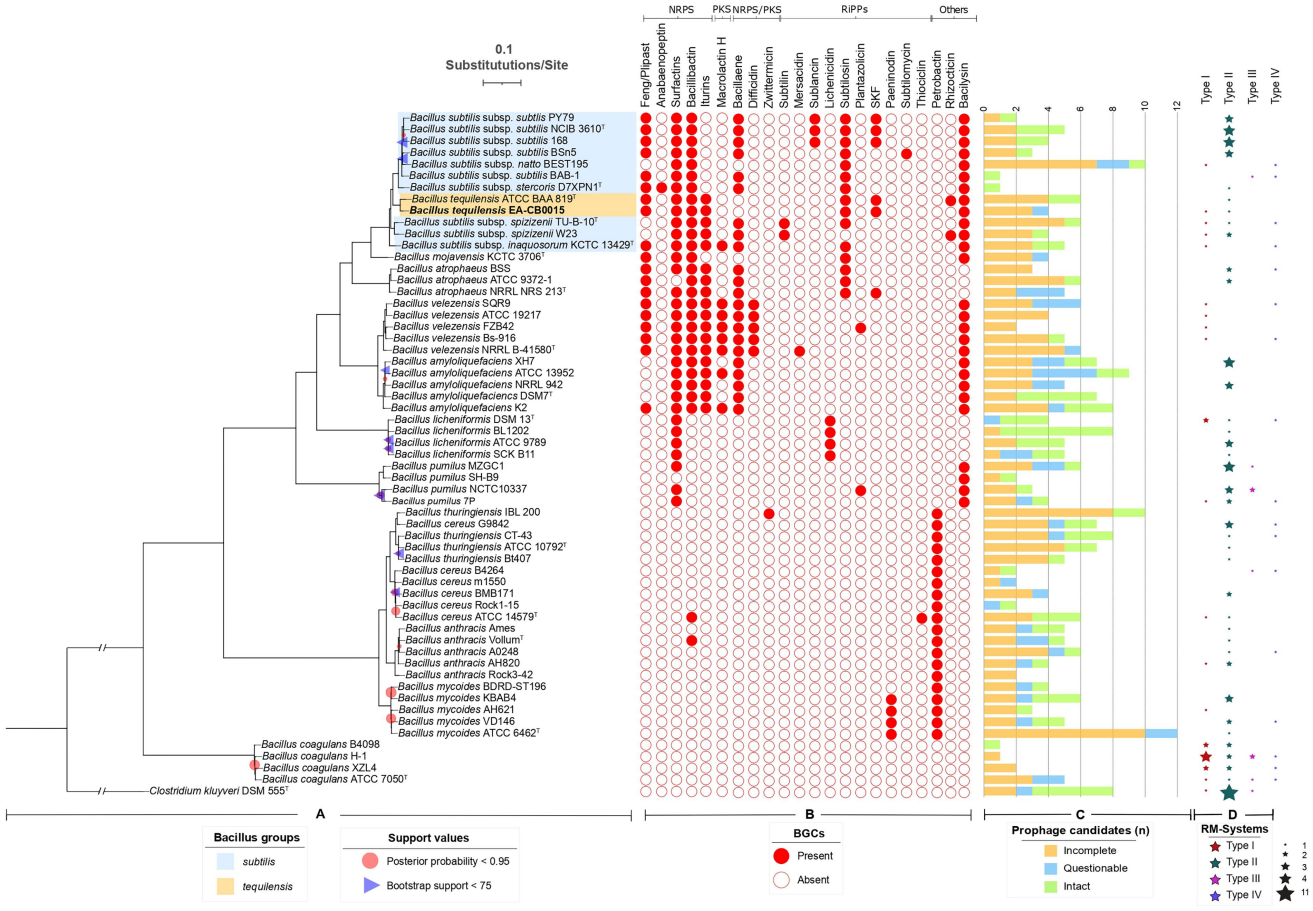
Phylogeny of EA-CB0015 and related *Bacilli* with their natural product BGCs, prophages and RM systems. **(A)** Consensus MLSA phylogeny from maximum likelihood (ML) and Bayesian inference (BI). Node supports with posterior probability values <0.95 and bootstrap support values < 75 are shown. **(B)** Natural product BGCs predicted by antiSMASH. NRPs, non-ribosomally synthetized peptides; PKs, polyketides; RiPPs, ribosomally produced post-translationally modified peptides. **(C)** Number of regions annotated as prophages according to PHASTER. Green bars indicate intact prophages (scores > 90); blue bars, questionable (70–90); orange bars, incomplete (<70). **(D)** Distribution and abundance of RM-Systems according to REBASE or manually searched.

EA-CB0015 was originally identified as *B. subtilis* (Ceballos et al., 2012) and later referred to as *B. tequilensis* (Cuellar-Gaviria et al., 2021). This ambiguity derived from the use of 16S rRNA gene phylogenies, which do not provide sufficient resolution at the species level for strains within the *B. subtilis* complex (Rooney et al., 2009). To definitively determine the species identity of the strain, we performed multilocus sequence analysis (MLSA) using established marker genes shown to effectively determine speciation in *Bacillus*. EA-CB0015 was more closely related to *B. tequilensis* ATCC BAA 819^T^ than to other *B. subtilis* subspecies, including *subtilis*, *natto*, *stercoris*, *spizizenii* and *inaquosorum* (Figure 2A). This is supported by high ANI values between the genomes of strain EA-CB0015 and *B. tequilensis* ATCC BAA 819^T^ (98.6%), vs. *B. subtilis* subsp. *inaquosorum* KCTC 13429^T^ (92.3%) *B. subtilis* subsp. *subtilis* 168 (91.4%; Supplementary Table S2). Thus, EA-CB0015 belongs to the phylogenetically homogeneous *B. subtilis* species complex and is classified as *B. tequilensis*.

### Comparative genomics of *Bacillus tequilensis* EA-CB0015, *Bacillus tequilensis* ATCC BAA 819^T^, and *Bacillus subtilis* 168

**Figure 3:**
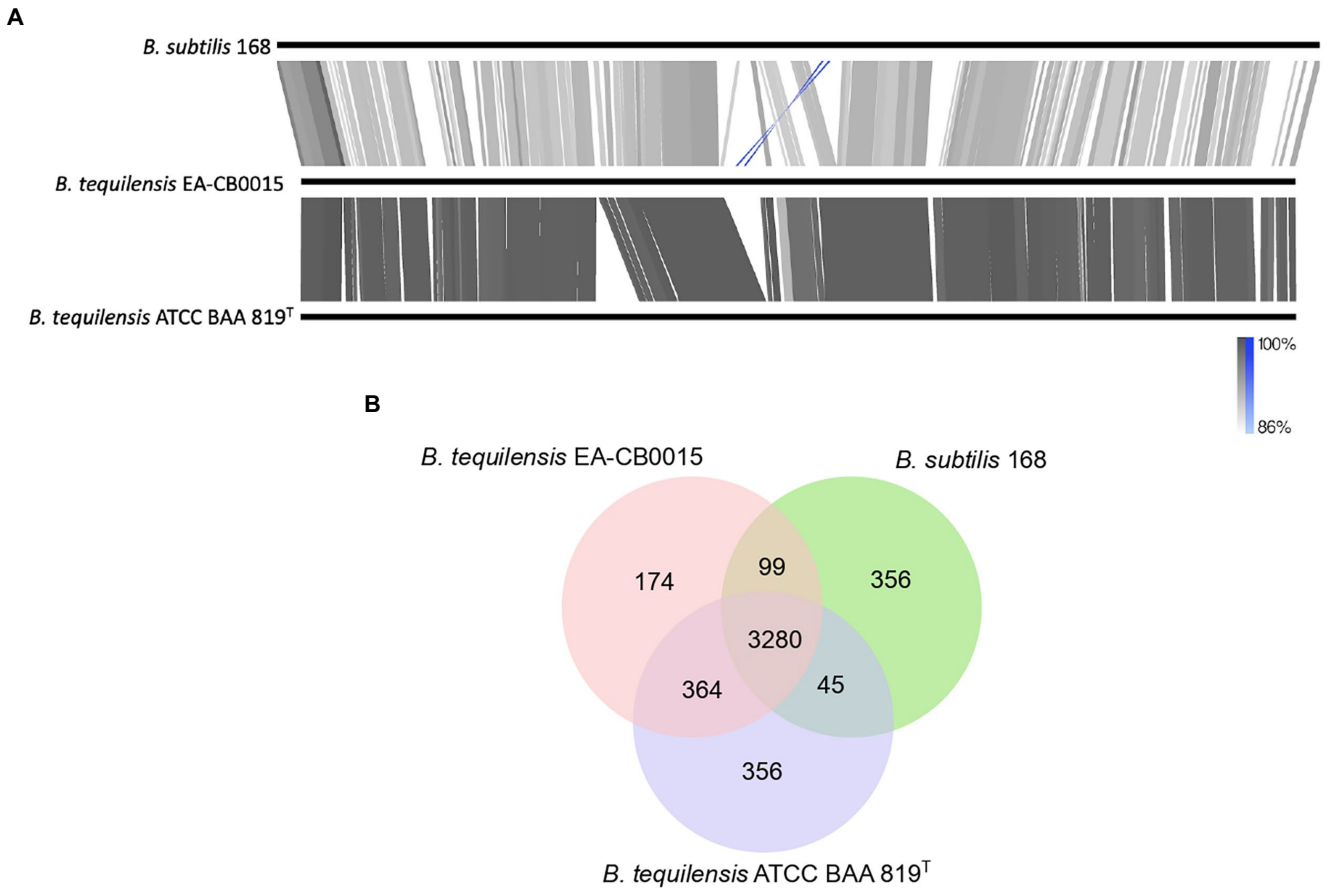
Comparison of *Bacillus subtilis* 168, *Bacillus tequilensis* EA-CB0015 and *B. tequilensis* ATCC BAA 819^T^ **(A)** Global genome alignments. Gray lines and blocks indicate regions and degree of shared similarity. Blue represents inversions **(B)** Shared and unique proteins between the three strains.

The phylogenetic analysis suggests *B. tequilensis* and *B. subtilis* share a close evolutionary history. To understand the nature of this relationship, we identified genetic similarities and differences between EA-CB0015, ATCC BAA 819^T^, and 168. The GC content of all three strains were similar (43.5%–44.0%) and they contained the identical number of rRNA and tRNA encoding genes. The *B. tequilensis* genomes were comparable in size (4 Mbp) and the number of protein coding sequences, but both were smaller than *B. subtilis* 168 (4.2 Mbp; Supplementary Table S3). Except for an extra 1.4 kbp plasmid in ATCC BAA 819^T^, both *B. tequilensis* genomes were largely syntenic (Figure 3A). Although less conservation was observed between 168 and EA-CB0015, large segments of their genomes remained homologous with each other. A 132 kbp inversion corresponding to bacteriophage SPβ was near the replication terminus (*terC*) of both strains (Figure 3A). In strain 168, the prophage was encoded on the complementary strand 134 kb upstream *terC*, while in strain EA-CB0015 it was on the positive strand 186 kb downstream of *terC*. Interestingly, this region was highly variable between 168 and EA-CB0015, and completely absent from the genome of *B. tequilensis* ATCC BAA 819^T^. Compared to 168, the SPβ prophage region of EA-CB0015 lacked the sublancin BGC and its immunity gene (Denham et al., 2019), a gene encoding for DNA cytosine-5-methyltransferase (*mtbP*), and numerous hypothetical proteins with unknown function. Most of the encoded proteins and their families were shared between all three strains (Figure 3B; Supplementary Table S4).

### Motility and biofilm formation

Multicellular behavior is used by *B. subtilis* to afford survival in its natural environment. These mechanisms include motility, biofilm formation, cannibalism, quorum sensing, competence, and sporulation (González-Pastor, 2017). As *B. subtilis* 168 is well studied for its ability to assemble multicellular communities and closely related to EA-CB0015 (O’Toole et al., 2000; González-Pastor, 2017), we examined if genes for these phenotypes were also present in our strain.

Genes for flagellum-mediated motility including chemotaxis and swarming were present in the EA-CB0015 genome. All thirty-two genes required to form the flagellar basal body and hook were encoded within a 27-kb *fla/che* operon, as well as all the genes needed for filament formation (flagellin monomer protein Hag, FliD, FlgK, FlgL), assembly, and rotation (Supplementary Table S5A; Mukherjee and Kearns, 2014). Other genes required for the basal body formation (FlhO and FlhP), torque in flagellar rotation (MotA, MotB), cell separation (LytC), bistable regulation (SigD, SwrA, SwrB, DegS/DegU, YmdB, and SlrA/SinR/SlrR), chemotaxis and associated chemoreceptors (2 soluble and 7 membrane-bound) were also present (Supplementary Tables S5A,B). Microscopy showed motile, polarly flagellated cells (Supplementary Figure S3). These results suggest EA-CB0015 can sense and swim toward a wide range of attractants (pH, amino acids, oxygen, etc.) that are present in its local environment.

To form a biofilm, cells must transition from motile to sessile states, aggregate, and embed themselves within a self-produced extracellular matrix (EM; Arnaouteli et al., 2021). EA-CB0015 formed biofilms in static culture (Supplementary Figure S3), and as expected, the genome of EA-CB0015 encoded genes essential for this process including exopolysaccharide biosynthesis (*epsA-O*), protein fiber TasA (*tapAsipWtasA* operon), the hydrophobin-like protein BslA, and genes for poly-γ-glutamic acid (γ-PGA) biosynthesis (*pgsBCAE*; Supplementary Table S5C). Production of EM is also linked to multiple regulatory proteins that were all present within the genome (Supplementary Table S5C). The Spo0A pathway (KinC, KinD, Spo0A, SinI/SinR/SlrR, AbrB) controls the expression of more than 100 genes including biofilm matrix gene expression and sporulation, while the YwcC-SlrA stress response pathway facilitates adaptation to changing environmental conditions. The DegS–DegU two-component system regulates competence, motility and secretion of degradative enzymes. Lastly, genes that mediate expression of *slrR*, an essential regulator of biofilm formation were also present (*abh*, *ymdB*, *remA*, *remB*; Vlamakis et al., 2014).

Interestingly, EA-CB0015 does not harbor the *yitPOM* operon. The paralogous *sdpABC* operon, is also absent from the genome of the strain. The *yitPOM* operon expresses the biofilm associated toxin (YitM) and the extracellular protease (NprB), both of which suppress competitors in *B. subtilis* biofilms (Kobayashi and Ikemoto, 2019). In addition, surfactin, encoded in the *srfA* operon (Supplementary Table S5B), may induce potassium leakage that stimulates the sensor kinase KinC. This may also activate expression of biofilm formation genes (López et al., 2009).

### Cannibalism

Cannibalistic behavior delays the entry into sporulation within a subpopulation of cells. This is controlled by the production of sporulation delaying protein-Sdp and sporulation killing factor-Skf, which lyse and kill sensitive siblings (González-Pastor et al., 2003). Interestingly, EA-CB0015 contained genes for Skf (*skfA-H*), but not Sdp (*sdpABC* and *sdpRI* operons were absent; Supplementary Table S5D). These results suggest EA-CB0015 cells may exhibit an accelerated sporulating phenotype. As Spo0A-inactive cells are not lysed, the pool of nutrients released into the environment from cell death is reduced. Thus, neighboring cells experience starvation to initiate spore formation (González-Pastor et al., 2003).

### Quorum sensing and competence

Genes encoding mechanisms of quorum sensing were present in the EA-CB0015 genome (Supplementary Table S5E). These may allow strain EA-CB0015 to coordinate physiological processes such as the synthesis of exoproteases and other extracellular enzymes in response to cell density. The lipopeptide surfactin is also positively regulated by the phosphorylated form of ComA, which is part of the quorum sensing (QS) system (ComQXPA) in *B. subtilis* (Nakano et al., 1991; Kalamara et al., 2018). Interestingly, the putative ComQ, ComX, and ComP proteins shared low sequence identity with their corresponding homologs in 168 (Supplementary Table S5E). This suggests the Com system may handle separate social communication groups or pherotypes (Stefanic and Mandic-Mulec, 2009; Oslizlo et al., 2014). The genome also encoded six putative receptor-signal pairs of the Rap-Phr system (Rap-Phr A, C, E, F, H; Supplementary Table S5E). Similar to 168, accumulation of Phr peptides in EA-CB0015 may suppress effects of Rap proteins to allow expression of genes for swarming motility, biofilm formation, exoprotease secretion and genetic competence (Kalamara et al., 2018). Related to quorum sensing, EA-CB0015 also contained genes for the acquisition and incorporation of extracellular DNA into the host cell (genetic competence). These include the master competence regulator *comK*, genes encoding proteins essential for DNA binding and import (*comC*, operons *comE*, *comF*, *comG, bdbD, bdbC*), and cytosolic proteins that modulate recombination and transformation efficiency (RecA, SsbB, DprA, CoiA, NucA). Genes encoding proteins for transcriptional (Rok, CodY, Kre) and post-translational regulation (MecA, ComS) of ComK were also present (Supplementary Table S5F).

### Sporulation

Endospores were visible when cells were subjected to Ryu stain (Supplementary Figure S3). Indeed, genes needed for sporulation were present in the genome (Supplementary Table S5G). These included genes encoding for morphogenetic proteins (SpoIVA, SpoVM, SpoVID, SafA, CotE, CotX/CotY/CotZ), their interacting partners during spore coat assembly, and spore crust proteins. Interestingly, some shared < 60% sequence identity with the homologs in 168, while others were completely absent (Supplementary Table S5G). The biosynthetic genes for legionaminic acid, which is used in crust formation, were notably missing (Supplementary Table S5G). Their absence suggests that the surface of EA-CB0015 spores may be reduced in hydrophilicity and charge (Dubois et al., 2020).

Most of the genes encoding signal transduction proteins (histidine sensor kinases KinA-E, master regulator Spo0A, phosphotransferases Spo0F and Spo0B, etc) related to sporulation were identified (Supplementary Table S5G). Homologs of *lrpAB* were missing, but other studies have indicated negligible effects on *glyA* transcription or sporulation through KinB (Dartois et al., 1997). The absence of *sivC* may result in a greater sporulation efficiency, as it functions as an inhibitor of the KinB and KinC pathway (Garti-levi et al., 2013). As expected, genes encoding proteins involved with major events in spore gemination were also present (Setlow et al., 2017). These include genes needed for germinant sensing (germinant receptors), release of dipicolinic acid (DPA) related to heat resistance (spoVA proteins, GerD), and hydrolysis of cortex peptidoglycan (CwlJ, SleB, SleL; Supplementary Table S5G). Altogether, these data suggest EA-CB0015 endospores can fully germinate once in favorable environmental conditions.

### Pathways for carbon assimilation

Nutrient availability on leaves, especially for organic compounds, is spatially heterogeneous and limited (Leveau and Lindow, 2001; Lindow and Brandl, 2003). A major carbon source that leach from the interior of the plant are common sugars (Wildman and Parkinson, 1981; Fiala et al., 1990; Lindow and Brandl, 2003). EA-CB0015 encodes all the genes necessary for glucose assimilation into the TCA cycle (KEGG pathway map ID bteq00020) by glycolysis (bteq00010). Likewise, genes for the metabolism sucrose and starch (bteq00500), and fructose (*via* fructose-1P and frutokinase (bteq00051)) were present. Pathways for galactose, maltose, and raffinose catabolism were also predicted (bteq00052, bteq00500). However, not all the genes needed for inositol utilization were present (bteq00562), such as those that encode transporters (*iolF, iolT*). Genes needed for scyllo-inosose transformation (*iolE, iolD, iolB, iolC, iolJ*) were also absent (Supplementary Table S6).

In addition to sugars, organic acids (e.g., l-lactic acid, citric acid, and l-malic acid) are common on the foliage of plants (Morgan and Tukey, 1964). EA-CB0015 utilized these and other compounds as carbon sources in API 50 CHB/E (Villegas-Escobar et al., 2013) and Biolog GEN III Microplate growth assays (Supplementary Table S7). Although methanol is readily available on plant surfaces (Kutschera, 2007), the absence of genes for methanol dehydrogenase suggest EA-CB0015 is unable to consume it as carbon source. However, genes for formaldehyde fixation (*hxlA*, *hxlB*) shared high sequence identity (>90%) with their corresponding homologs in 168, suggesting formaldehyde may be assimilated through the ribulose monophosphate pathway (bteq_M00345, Supplementary Table S6). This may allow EA-CB0015 to scavenge formaldehyde produced by neighboring methylotrophs during growth on plant surfaces.

### Hydrolytic enzymes

EA-CB0015 contained genes for β-glucanases (*bglC*, *bglS*), chitinase (*Csn*), extracellular proteases (*aprE*, *nprE*), bacillopeptidase F protein degradation (Bpr), endolevanase (*levC*), xylanase (*xynA, B, and C*), α-amylase (*amyE, amyX*), and pectate lyases (*pel*, *pelB*; Supplementary Table S8). These enzymes are be used to liberate sugars from complex polysaccharides that are then be assimilated by the strain. Chitinase may also function to inhibit nearby fungi by hydrolyzing their cell walls and inhibiting formation of hyphae (Kobayashi and Ikemoto, 2019; Legein et al., 2020). Lastly, a gene encoding for the quorum quenching enzyme YtnP (lactonase-homolog protein) was also found in the genome. This may degrade quorum sensing autoinducers of competitive strains in the same plant environment (Schneider et al., 2012).

### Nitrogen and phosphorous

EA-CB0015 contained genes required to reduce nitrate to ammonia *via* the dissimilatory nitrate reduction pathway (bteq_M00530, Supplementary Table S7). The strain lacks genes for a nitrogenase enzyme complex, and thus is unable to fix atmospheric dinitrogen. Genes required for metabolism of aspartate/glutamate (bteq00250), arginine/proline (bteq00330), glycine/threonine/serine (bteq00260) were present (Supplementary Table S7), all of which have been detected in the foliage of plants (Morgan and Tukey, 1964; Parangan-Smith and Lindow, 2013).

Under phosphate limiting conditions, many microorganisms can solubilize inorganic sources of phosphate or mineralize organophosphorous compounds (Hanif et al., 2015). Genes encoding for the *pho* regulon, including alkaline phosphatase, extracellular enzymes to catabolize organophosphorous compounds (PhoA, PhoB, PhoD, GlpQ), phosphate transporters, and the *tat* secretion system for protein export were indeed present within the genome (Supplementary Table S9; Allenby et al., 2005). Interestingly, phytase (*phy*) was only present as a pseudongene (Supplementary Table S9), suggesting strain is unable to hydrolyze phytate, a major source of organic phosphate.

### Iron

EA-CB0015 encoded genes for the biosynthesis of the siderophore bacillibactin (*dhbACEBF*), transporters for importing it into the cell (FeuABC-YusV), and the esterase BesA to release the iron in the cytosol (Supplementary Table S10; Miethke et al., 2006). Genes encoding hydoxamate (*fhuBCDG*), petrobactin/catecholate (*fpbOPQ*), schizokinen/anthrobactin (*yfhAYZ*) transporters suggest the strain may scavenge siderophores produced by other species (Supplementary Table S10). Genes for citrate-iron transporter (*fecCDEF*), high-affinity iron transporter (*efeUOB*), and heme degradation (*hmoAB*) suggest these as additional mechanisms for iron acquisition.

### Protective mechanisms against oxidative stress and UV

Residing on plant leaves and surfaces, epiphytes are continually challenged by photooxidative stress. In addition to sporulation, biofilm formation, and motility, EA-CB0015 contained genes to protect itself from desiccation, UV light, and oxidative damage (Mols and Abee, 2011). The flavin-dependent photoreceptor (*ytvA*) activates general stress response mechanisms in the presence of blue light. This includes spore-product photolyase (*splB*; Supplementary Table S11) to repair thymine dimer adducts produced from UV radiation (Herrou and Crosson, 2011; Vanhaelewyn et al., 2020). Genes for catalase, superoxide dismutase, thioredoxin reductase, hydroperoxide reductase, peroxiredoxin, and antioxidants including bacillithiol, phytoene, and sporulene (Supplementary Table S11), may help EA-CB0015 reduce damage from oxidative stress (Engelmann and Hecker, 1996; Newton et al., 2009; Zuber, 2009; Jeong et al., 2018). Production of extracellular polysaccharides and the siderophore bacillibactin (a UV-B absorbing compound) may further allow EA-CB0015 to avoid damage from UV exposure (Supplementary Table S11).

### Biosynthetic pathways for natural products

**Figure 4:**
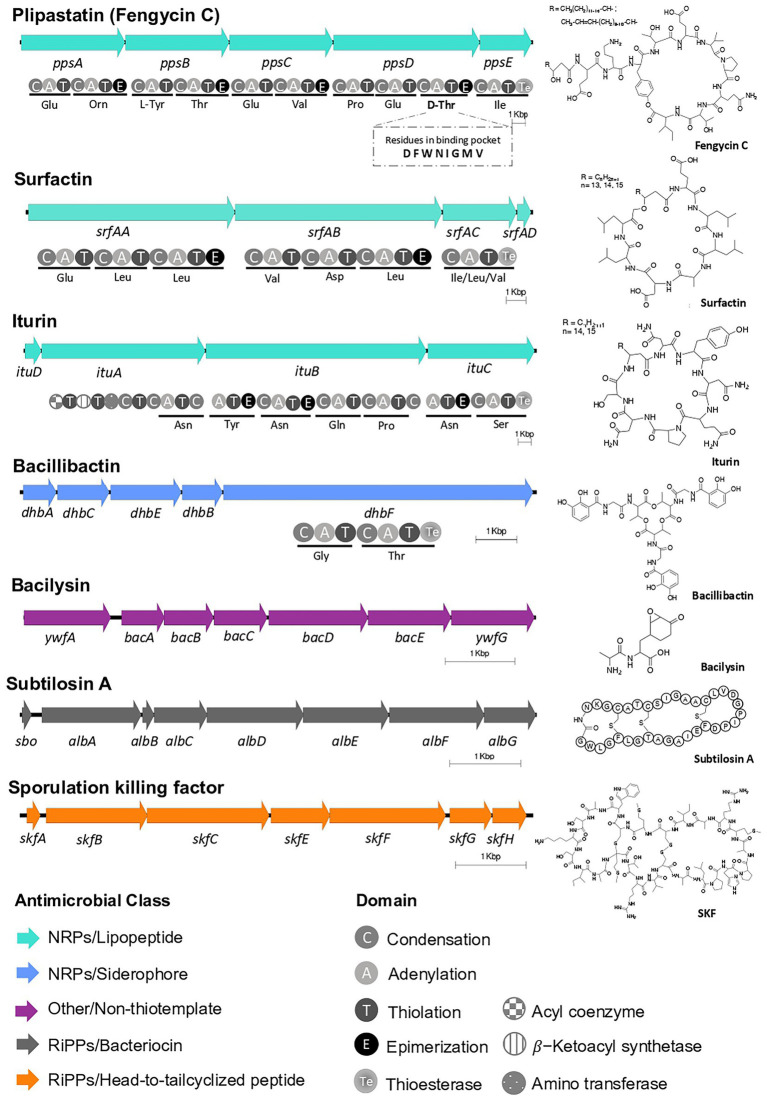
BGCs of known natural products within *Bacillus tequilensis* EA-CB0015. Predicted domains for NRPSs and amino acid specificity are shown. The third adenylation domain in the putative PpsD protein is predicted to load threonine (fengycin C) instead of tyrosine (plipastatin B). The BGCs are located on the following positions on the chromosome: SKF (209,791–215,884); surfactin (359,914–386,063); iturin* (1,919,24–1,955,499); plipastatin (fengycin C)* (1,969,256–2,007,030); bacillibactin* (3,075,812–3,087,611); subtilosin A* (3,667,130–3,674,071); bacilysin* (3,701,710–3,708,994). (*Encoded on negative strand).

Epiphytic bacteria scavenge nutrients, influence development of plants, and the composition of local microbial communities through their natural products (Legein et al., 2020). To identify the repertoire of molecules EA-CB0015 may produce, we first analyzed its genome using antiSMASH. We found seven BGCs for known natural products and two of unknown function (Supplementary Table S12). Encoded within the genome were non-ribosomal peptide synthetase (NRPS) BGCs for lipopeptides (plipastatin/fengycin C, surfactin, iturin) and the siderophore bacillibactin. The BGCs for subtilosin A (thiopeptide), sporulation killing factor (SKF; sactipeptide), and bacilysin were also detected (Figure 4). Annotation of the NRPS domains for fengycin C (Villegas-Escobar et al., 2013), suggests the strain actually produces plipastatin. Specifically, the lack of an epimerization domain in module 3 of *fenB* suggests incorporation of l-Tyr instead of d-Tyr. Moreover, the domain annotation of module 9 predicts epimerization, suggesting d-Thr instead of a l-Thr in the final peptide natural product (Hussein, 2019). The neighborhoods from EA-CB0015 were all conserved to reference BGCs for these natural products (Supplementary Figure S4).

Two genomic regions were annotated to encode for potential terpene and polyketide compounds. The first region (2,107,965 to 2,129,124 bp) contained a gene annotated as squalene-hopene cyclase, but its neighborhood lacked commonly associated genes for condensation of farnesyl diphosphate to squalene (SQase, or *hpnD*, *hpnC*, *hpnE*; Supplementary Table S13; van der Donk, 2015). The second region (2,174,022 and 2,213,530 bp) contained a putative type III polyketide synthase (chalcone synthase; BpsA) and an isoprenylcysteine carboxyl methyltraferase (BpsB), suggesting a role for biosynthesis of aliphatic polyketides, such as triketide pyrones, tetraketide pyrones and alkylresorcinols (Supplementary Table S14; Nakano et al., 2009).

Putative pathways for other natural products were identified by KEGG and manual annotation. EA-CB0015 encoded genes for the antibiotic kanosamine, which is biosynthesized in three steps from glucose-6-phosphate (enzymes NtdC, NtdA, NtdB; KEGG accession bteq00998, Supplementary Table S8). Biosynthesis of the plant hormone IAA included genes for tryptophan aminotransferase (*patB*), idole-3-pyruvate decarboxylase (*yclC*), and indole-3-acetaldehyde dehydrogenase (*dhaS*). These would produce indole 3-pyruvic acid pathway (IPyA) the predominant precursor to IAA (Shao et al., 2015). Genes related to the tryptamine-TAM pathway (*bsdC,* flavin monamine oxidase, and *dhaS*), known as an alternative pathway for the synthesis of IAA, were found (Shao et al., 2015). *YhcX,* predicted to act as a nitrilase in the last step of the indole 3-acetonitrile (IAN) pathway, was also present (Supplementary Table S15).

### Genetic exchange

Leaf surfaces are proposed as hot spots for lateral gene transfer and important breeding grounds for microbial diversity (Lindow and Leveau, 2002). Thus, we sought to understand both the degree to which EA-CB0015 may have been affected by gene transfer events and mechanisms it may have to maintain genome integrity.

**Figure 5:**
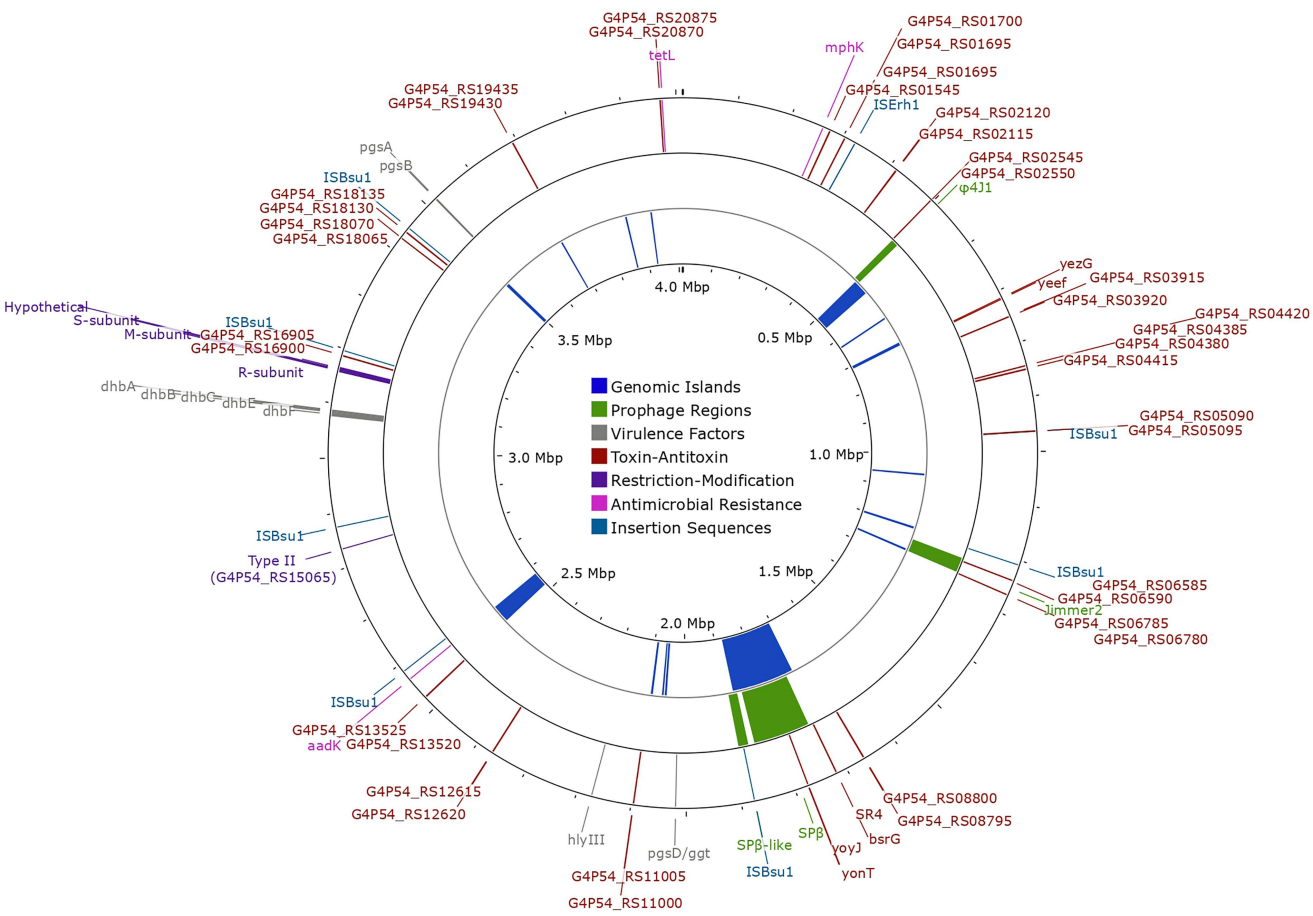
Chromosomal map of *Bacillus tequilensis* EA-CB0015 showing genetic exchange features, antimicrobial resistance genes, restriction modification systems, and virulence factors. The outer circle shows the location of insertion sequences (light-blue), antimicrobial resistance genes (pink), restriction modification systems (purple), toxin-antitoxin systems (brown) and virulence factors (gray). Intact (high confidence) prophage regions are shown in the second circle (green). The inner circle shows the location of genomic islands (dark-blue).

EA-CB0015 encoded genes needed to take up DNA by genetic competence (Supplementary Table S5), but did not contain plasmids nor any complete integrative or conjugative transposons (Auchtung et al., 2016). However, some pseudogenes (*immA*, *immR*, *phrI*) or integrases (*int*) for excision of ICEBs were present in the genome (Supplementary Table S16). Four prophage regions were predicted, suggesting EA-CB0015 was susceptible to bacteriophages (Figure 5; Supplementary Figure S5). PHASTER analysis classified three as incomplete (*Bacillus* phi4J1, *Brevibacillus* Jimmer2, and *Staphylococcus* SPβ-like) and one as questionable (*Bacillus* SPβ). While insertions and recombination from prophages may disrupt important genes, they may also introduce phage resistance or prototrophy to improve the competitive fitness (Kohm and Hertel, 2021).

Other hallmarks of horizonal gene transfer include GEIs and transposable elements. GEIs encode cluster of genes for specialized functions (pathogenicity, symbiosis, metabolism, resistance, fitness) presumed to be of foreign origin (Juhas et al., 2009). Interestingly, GEIs accounted for 10% of the total genome, with many phage-related genes overlapping the same regions (Figure 5; Supplementary Figures S5, S6). Fourteen ISs (transposable elements) were present in the genome (Supplementary Table S17). However, ISs belonging to IS1182 and IS1595 families were pseudogenes and only those belonging to the IS3 family were complete. The latter was the most abundant, with 8 copies of ISBsuI and 1 copy of ISErh (Siguier et al., 2006; Figure 5). These may offer a selective advantage by accelerating genome rearrangement, introducing virulence factors, or resistance genes to antibiotics (Lindow and Leveau, 2002). Genes for putative virulence factors including hemolysin III (*hlyIII*), capsular polyglutamate and bacillibactin were encoded outside of mobile elements (Figure 5; Supplementary Table S18). Resistance genes for streptomycin (*aadK*), spiramycin and telithromycin (*mphK*), and tetracycline (*tetL*; Figure 5; Supplementary Tables S18, S19) were also detected. The streptomycin resistance gene was encoded within a genomic island.

Both type I and type II RM systems were encoded in the genome of EA-CB0015. The type I system contained genes for the HsdR endonuclease (R), specificity subunit (S), and DNA methyltransferase (M; Figure 5; Supplementary Figure S7). While genes for the endonuclease and methyltransferase of the type I system were highly conserved (>85% sequence identity), the specificity subunit (G4P54_RS16760) exhibited significantly lower sequence identity (33%–40%; Supplementary Figure S8). This suggests variability in the recognition site where the restriction endonuclease cleaves DNA. Additionally, a type II RM system with a site-specific DNA methyltransferase (locus tag G4P54_RS15065) was found.

EA-CB0015 contained 22 TA systems (Figure 5; Supplementary Figure S9; Supplementary Table S20). These included two type I TA systems (*bsrG/SR4* and *yonT-yoyJ/SR6* located in SPβ region) and three type II TA systems (*ndoA/ndoB, spoIISA/spoIISB/spoIISC* and *yeeF/yezG*) which are also in strain 168. TA systems may provide fitness advantages when the strain experiences stress from pH changes, oxygen deficiency, or iron limitation (Brantl and Müller, 2019). Lastly, no RNA-based defense systems were found using CRISPRCasFinder (Couvin et al., 2018).

### Natural product BGCs, prophages and restriction modification systems among *Bacillus* spp.

To understand how EA-CB0015 compares with these closely related bacteria and the presence of any distinguishing trends, we cataloged natural product biosynthesis, prophage, and RM across type strains within the *B. subtilis* species complex, *B. cereus* species complex and *B. coagulans* (Figure 2). In general, strains that are non-pathogenic to humans (*B. subtilis*, *B. tequilensis*, *B. mojavensis*, *B. atrophaeus*, *B. velezensis*, *B. amyloliquefaciens*, *B. licheniformis* and *B. pumilus*) encoded a more natural product BGCs. Among the 22 different natural product BGCs we identified, the largest number (7–9) were associated with *B. subtilis*, *B. velezensis*, *B. amyloliquefacies*, and *B. tequilensis* (Figure 2B). These was consistent with known biological control agents produced by these species (Chen et al., 2009; Gutierrez-Monsalve et al., 2015; Pandin et al., 2018; Samaras et al., 2021). Surfactin, bacilysin, bacillibactin, and bacillaene were the most prevalent BGCs. Except for the antifungal phosphonopeptide rhizocticin (Borisova et al., 2010), EA-CB0015 and BAA-819 share the same composition of natural product BGCs. The most common BGC in pathogenic bacilli was petrobactin. None of these 22 BGCs were present in *B. coagulans*.

Prophages account for substantial genetic variation and confer phage resistance (Casjens, 2003; Fortier and Sekulovic, 2013; Kohm and Hertel, 2021). Across all taxa, the number of candidate prophage regions and prophage CDSs was similar within the *B. subtilis* species complex (4.68 regions and 217.74 CDSs) and *B. cereus* group (5.15 regions and 196.95 CDSs; Figure 2C; Supplementary Figure S10). Intact prophages included members of the Siphoviridae, Myoviridae and Tectiviridae families (Supplementary Figure S11). Twelve phages were unique to *B. subtilis* species complex, 8 unique to the *B. cereus* species complex, and 3 were shared between both groups (Supplementary Figures S10, S12). Altogether, these results indicate phages are evolutionary conserved within species complexes.

As natural selection is one of the most important evolutionary processes, RM systems serve as an important defense against the introduction of foreign DNA (Bourniquel and Bickle, 2002). Type II RM systems were the most common among both groups, present in 68 and 81% of the strains from *B. subtilis* and *B. cereus* complexes, respectively. Other types of RM systems were also widely distributed but less abundant than type II systems (Figure 2D), but no differential patterns were observed between these two groups of strains.

## Discussion

Successful biocontrol agents have been suggested to require distinct mechanisms that confer survival to its habitat and contribute to the health of its host (Legein et al., 2020). In sequencing the genome of *B. tequilensis* EA-CB0015, we uncovered the genetic basis for several physiological adaptations underlying its survival in the phyllosphere including biofilm formation, motility, competence, protection from oxidative stress, and sporulation. Spores of EA-CB0015 are predicted to lack legionaminic acid, and their reduced charge may favor adherence to the naturally hydrophobic surface of leaves. Genes for chemotaxis, and swarming suggest colonization of plant surfaces may be mediated through motility toward optimal growth conditions including microenvironments rich in nutrients. EA-CB0015 may also leverage acquisition of nutrients through its biosynthesis of IAA and surfactin. Production of IAA induces physiological changes in plants including loosening of plant cell walls and the release of nutrients (Vanderhoef and Dute, 1981). Surfactin could improve the wettability of leaves and thus facilitate adherence (Lindow and Brandl, 2003). Both may increase access of nutrients that could then be assimilated by EA-CB0015 through its diverse pathways for carbon (sugars, organic acids, formaldehyde) nitrogen (dissimilatory nitrate reduction, amino acid catabolism), and phosphorous assimilation.

Microbial natural products contribute the health of host plants by modulating plant hormone concentrations, inducing systemic resistance, and inhibiting both growth and intercellular communication of pathogens (Legein et al., 2020). At least seven natural product BGCs were present within the genome of EA-CB0015. In addition to previously characterized lipopeptides surfactin, iturin A and fengycin C (Villegas-Escobar et al., 2013; Mosquera et al., 2014), four additional natural product BGCs for bacillibactin, bacilysin, subtilosin A, and the sporulation killing factor were identified. These later compounds could contribute to the biological control activities of this strain. In addition to iron scavenging and biofilm formation, bacillibactin has been associated with alternative functions including transport of other metals, sequestration of toxic metals, and protection from oxidative stress (Rizzi et al., 2019; Legein et al., 2020). Production of bacilysin by *B. velezensis* FZB42 was found to regulate the expression of several virulence genes in *X. oryzae* (Özcengiz and Öğülür, 2015; Wu et al., 2015), while subtilosin A was inhibitory against a variety of gram-positive and-negative bacteria (Shelburne et al., 2007). The cannibalistic peptide, SKF is known to permeabilize of cytoplasmic membranes of *E. coli* cells (Nonejuie et al., 2016) and inhibit the growth of plant pathogens such as *X. oryzae* (Lin et al., 2001). Determining the production of these compounds by EA-CB0015 on plants will provide valuable insight into their functional role in biocontrol and if beneficial synergistic effects may exist.

We identified significant variations between related *Bacilli* that arose from bacteriophage activity. Long-term associations between temperate phages may provide benefit to bacteria through resistance against infection and introduction of accessory genes for metabolism, stress tolerance, and antibiotic resistance. These may facilitate survival of EA-CB0015 when exposed to natural products produced from other epiphytic microbes and antibiotics commonly applied to commercial crops (Schnabel and Jones, 1999; Feiner et al., 2015; Howard-Varona et al., 2018; Ramisetty and Sudhakari, 2019). Moreover, mobile genetic elements in EA-CB0015 and related *Bacilli* may accelerate transfer of genes that are advantageous for their survival in the phyllosphere (Lindow and Leveau, 2002). The contribution of phages, mobile elements, and associated TA systems on the competitive fitness of epiphytic *Bacillus* spp., influencing the phyllosphere microbiome, and effecting the physiology of host plants remain important areas of future investigation.

## Data availability statement

The original contributions presented in the study are publicly available. This data can be found at: https://www.ncbi.nlm.nih.gov/nuccore/NZ_CP048852.1.

## Author contributions

TC-G, K-SJ, and VV-E: conceptualization and writing the manuscript. TC-G, CG-B, K-SJ, and VV-E: methodology, investigation, and formal analysis. K-SJ and VV-E: supervision and funding acquisition. All authors contributed to the article and approved the submitted version.

## Funding

This project was funded with support from Universidad EAFIT (VV-E), Association of Banana Producers of Colombia (AUGURA; VV-E), Colciencias (Convocatoria 617 Doctorados Nacionales for the PhD studies of TC-G), and the National Institutes for Health (GM137135 to K-SJ).

## Conflict of interest

The authors declare that the research was conducted in the absence of any commercial or financial relationships that could be construed as a potential conflict of interest.

## Publisher’s note

All claims expressed in this article are solely those of the authors and do not necessarily represent those of their affiliated organizations, or those of the publisher, the editors and the reviewers. Any product that may be evaluated in this article, or claim that may be made by its manufacturer, is not guaranteed or endorsed by the publisher.

## Footnotes

^1^ https://github.com/camilogarciabotero/PHASTER-DataProcessing-scripts
