# Supplementary material for: The genome of Bacillus tequilensis EA-CB0015 sheds light into its epiphytic lifestyle and potential as a biocontrol agent
Source: Front Microbiol. 2023 Mar 27;14:1135487. doi: 10.3389/fmicb.2023.1135487 (PMC10083409; doi:10.3389/fmicb.2023.1135487)

Supplementary Information

The genome of *Bacillus tequilensis* EA-CB0015 sheds light into its epiphytic lifestyle and potential as a biocontrol agent

Tatiana Z. Cuellar-Gaviria[1,2], Camilo García-Botero[1], Kou-San Ju[2,3,4,5*], Valeska Villegas-Escobar[1*]

[1]CIBIOP Group, Department of Biological Sciences, Universidad EAFIT, Medellin, Colombia

[2]Department of Microbiology, The Ohio State University, Columbus, OH, USA

[3]Division of Medicinal Chemistry and Pharmacognosy, The Ohio State University, Columbus, OH, USA

[4]Center for Applied Plant Sciences, The Ohio State University, Columbus, OH, USA

[5]Infectious Disease Institute, The Ohio State University, Columbus, OH, USA

[*] **Correspondence:**

Valeska Villegas-Escobar and Kou-San Ju

Emails: vvilleg2@eafit.edu.co, ju.109@osu.edu

Table of Contents

Supplementary Tables -------------------- *3*

Table S1. *Bacillus* and *Clostridium* strains analyzed in this study --------------------3

Table S2. Average nucleotide identity (ANI) values between *B. tequilensis* EA-CB0015 and closely related *Bacillus* strains --------------------5

Table S3. Comparison of general genetic features between *B. tequilensis* EA-CB0015, *B. tequilensis* ATCC BAA 819^T and *B. subtilis* 168 --------------------5

Table S4. Unique genomic features in *B. tequilensis* EA-CB0015, *B. tequilensis* ATCC BAA 819^T and *B. subtilis* 168 *(see separate Excel file) --------------------5

Table S5. Multicellularity traits in *B. tequilensis* EA-CB0015*(see separate Excel file) --------------------6

Table S6. Carbohydrate and energy metabolism in *B. tequilensis* EA-CB0015 *(see separate Excel file)6

Table S7. Results of BioLog GEN III Microplate and API 50 CHB/E growth assays --------------------7

Table S8. Hydrolytic enzymes in *B. subtilis* 168 and *B. tequilensis* EA-CB0015 --------------------8

Table S9. Genes associated with phosphate metabolism from *B. subtilis* 168 and B. *tequilensis* EA-CB0015 -------------------- 10

Table S10. Genes associated with iron metabolism from *B. subtilis* 168 and B. *tequilensis* EA-CB001511

Table S11. Genes associated with UV and oxidative stress response -------------------- 15

Table S12. Natural product biosynthetic gene clusters present in the genome of *B. tequiliensis* EA-CB0015 -------------------- 20

Table S13. Annotation of putative terpene BGC neighborhood in *B. tequilensis* EA-CB0015 -------------------- 21

Table S14. Annotation of putative type III PKS BGC neighborhood in *B. tequilensis* EA-CB0015 -------------------- 23

Table S15. Indole-3 acetic acid (IAA) pathways identify in *B. tequilensis* EA-CB0015 -------------------- 26

Table S16. Genes associated with the integrative or conjugative element (ICEBs1) in *B. tequilensis* EA-CB0015 -------------------- 27

Table S17. Insertion sequences (ISs) in the genome of *B. tequilensis* EA-CB0015 -------------------- 29

Table S18. Genes for putative virulence factors -------------------- 29

Table S19. Predicted antimicrobial resistance genes in *B. tequilensis* EA-CB0015 -------------------- 30

Supplementary Figures -------------------- *33*

Figure S1. Cumulative GC skew for the genome of *B. tequilensis* EA-CB0015 -------------------- 34

Figure S2. Distribution of clusters of orthologous groups (COGs) for *B. tequilensis* EA-CB0015 -------------------- 35

Figure S3. Multicellular lifestyle traits of *B. tequilensis* EA-CB0015. -------------------- 36

Figure S4. Synteny of natural product BGCs from *B. tequilensis* EA-CB0015 against the reference sequences -------------------- 36

Figure S5. Prophages in the genome of *B. tequilensis* EA-CB0015 -------------------- 38

Figure S6. Circular view of the EA-CB0015 genome highlighting genomic islands (GIs) -------------------- 39

Figure S7. Type I restriction modification system identified from REBASE -------------------- 40

Figure S8. Top 14 results from cluster BLAST of the EA-CB0015 type I RM system -------------------- 41

Figure S9. Location of type II toxin/anti-toxin systems identified by TAfinder -------------------- 42

Figure S10. Comparisons of prophage regions between *B. subtilis* and *B. cereus* complex -------------------- 43

Figure S11. Diversity of intact prophage regions found in the genomes of different *Bacillus* species - 44

Figures S12. Venn diagram of intact phages reported for *B. subtilis* complex and *B. cereus* complex based on the MLSA -------------------- 45

Supplementary Tables

Table S1. *Bacillus* and *Clostridium* strains analyzed in this study

Species (Taxon name)	Strain	GenBank Assembly Accession
Bacillus tequilensis	EA-CB0015	GCA_012225885.1
Bacillus amyloliquefaciens	DSM 7^T	GCA_000196735.1
Bacillus amyloliquefaciens	ATCC 13952	GCA_000772125.1
Bacillus amyloliquefaciens	XH7	GCA_000221645.1
Bacillus amyloliquefaciens	K2	GCA_001866745.1
Bacillus amyloliquefaciens	NRRL 942	GCA_003546075.1
Bacillus anthracis	Ames	GCA_000007845.1
Bacillus anthracis	AH820	GCA_000021785.1
Bacillus anthracis	Rock3-42	GCA_000161235.1
Bacillus anthracis	A0248	GCA_000022865.1
Bacillus anthracis	Vollum^T	GCA_000742895.1
Bacillus atrophaeus	NRRL NRS 213^T	GCA_001584335.1
Bacillus atrophaeus	BSS	GCA_000742675.1
Bacillus atrophaeus	ATCC 9372-1	GCA_000204705.2
Bacillus cereus	ATCC 14579^T	GCA_000007825.1
Bacillus cereus	B4264	GCA_000021205.1
Bacillus cereus	G9842	GCA_000021305.1
Bacillus cereus	Rock1-15	GCA_000161175.1
Bacillus cereus	m1550	GCA_000161035.1
Bacillus cereus	IBL 200	GCA_000161715.1
Bacillus coagulans	ATCC 7050^T	GCA_000832905.1
Bacillus coagulans	XZL4	GCA_000223155.1
Bacillus coagulans	H-1	GCA_000333935.1
Bacillus coagulans	B4098	GCA_001587225.1
Bacillus licheniformis	DSM 13^T	GCA_000008425.1
Bacillus licheniformis	BL 1202	GCA_001726125.1
Bacillus licheniformis	SCK B11	GCA_002074075.1
Bacillus licheniformis	ATCC 9789	GCA_002504325.1
Bacillus mojavensis	KCTC 3706^T	GCA_000507105.1
Bacillus mycoides	ATCC 6462^T	GCA_000832605.1
Bacillus mycoides	KBAB4	GCA_000018825.1
Bacillus mycoides	AH621	GCA_000160975.1
Bacillus mycoides	BDRD-ST196	GCA_000161095.1

Bacillus mycoides	VD146	GCA_000399425.1
Bacillus pumilis	MZGC1	GCA_002998605.1
Bacillus pumilus	NCTC 10337^T	GCA_900186955.1
Bacillus pumilus	7P	GCA_000691485.2
Bacillus pumilus	SH-B9	GCA_001578205.1
Bacillus subtilis	NCIB 3610^T	GCA_002055965.1
Bacillus subtilis subsp. *inaquosorum*	KCTC 13429^T	GCA_003148415.1
Bacillus subtilis subsp. *natto*	BEST195	GCA_000209795.2
Bacillus subtilis subsp. *spizizenii*	TU-B-10^T	GCA_000227465.1
Bacillus subtilis subsp. *spizizenii*	W23	GCA_000146565.1
Bacillus subtilis subsp. *stercoris*	D7XPN1^T	GCA_000738015.1
Bacillus subtilis subsp. *subtilis*	168	GCA_000009045.1
Bacillus subtilis subsp. *subtilis*	PY79	GCA_000497485.1
Bacillus subtilis subsp. *subtilis*	BSn5	GCA_000186745.1
Bacillus subtilis subsp. *subtilis*	BAB-1	GCA_000349795.1
*Bacillus tequilensis**	ATCC BAA-819 ^{T}	N/A
Bacillus thuringensis	ATCC 10792^T	GCA_000161615.1
Bacillus thuringensis	CT-43	GCA_000193355.1
Bacillus thuringensis	Bt407	GCA_000161495.1
Bacillus thuringensis	BMB171	GCA_000092165.1
Bacillus velezensis	FZB42	GCA_000015785.1
Bacillus velezensis	NRRL B-41480^T	GCA_001461825.1
Bacillus velezensis	SQR9	GCA_000685725.1
Bacillus velezensis	ATCC 19217	GCA_000772165.1
Bacillus velezensis	Bs-916	GCA_000772205.1
Clostridium kluyveri	DSM 555^T	GCA_000016505.1

**Bacillus tequilensis* ATCC BAA-819™ = strain 10b = KCTC 13622T (GenBank Assembly Accession GCA_000507145.1). The genome sequence of this strain was downloaded from ATCC due to its quality and completeness.

Table S2. Average nucleotide identity (ANI) values between *B. tequilensis* EA-CB0015 and closely related *Bacillus* strains

Reference name	GenBank Accession	B. tequilensis EA-CB0015 ANI %
Bacillus tequilensis NCTC13306^T = ATCC BAA 819^T	GCA_900445435.1	98.6
Bacillus subtilis subsp. *inaquosorum* KCTC 13429^T	GCA_000332645.1	92.3
Bacillus subtilis subsp. *subtilis* 168	GCA_000009045.1	91.4
Bacillus amyloliquefaciens DSM 7^T	GCA_000196735.1	81.3
Bacillus licheniformis DSM 13^T	GCA_000008425.1	78.8

ANI values calculated using FastANI and GTDB-Tk.

Table S3. Comparison of general genetic features between *B. tequilensis* EA-CB0015, *B. tequilensis* ATCC BAA 819^T and *B. subtilis* 168

Attribute	***B. tequilensis* EA-CB0015**	***B. tequilensis* ATCC BAA 819^T**	***B. subtilis 168***
Genome size (bp)	4,012,371	4,018,158	4,215,606
Protein coding sequences	4,112	4,072	4,237
GC (%)	43.7	44.0	43.5
rRNA	30	30	30
tRNA	86	86	86
Plasmids	0	1	0

Table S4. Unique genomic features in *B. tequilensis* EA-CB0015, *B. tequilensis* ATCC BAA 819^T and *B. subtilis* 168 ***(see separate Excel file)**

A. Unique Gene Features *B. teq* EA15
B. Unique Gene Features *B. teq* ATCC
C. Unique Gene Features *B. sub* 168

Table S5. Multicellularity traits in *B. tequilensis* EA-CB0015***(see separate Excel file)**

A. Motility and chemotaxis
B. Swarming
C. Biofilm formation
D. Cannibalism
E. Quorum sensing
F. Genetic competence
G. Sporulation

Table S6. Carbohydrate and energy metabolism in *B. tequilensis* EA-CB0015 ***(see separate Excel file)**

A. Carbohydrate Metabolism
B. Energy Metabolism
C. Amino acid Metabolism

Table S7. Results of BioLog GEN III Microplate and API 50 CHB/E growth assays

Carbon Source	BioLog	API50	Carbon Source	BioLog	API 50	Carbon Source	BioLog	API50
Dextrin	+	ND	D-arabinose	ND	-	D-Serine	-	ND
D-Maltose ‡	+	+	Starch	ND	+	D-Alanine	+	ND
D-Trehalose ‡	+	+	Glycogen	ND	+	L-Arginine	+	ND
D-Cellobiose ‡	+	+	D-Turanose	ND	+	L-Aspartic acid	+	ND
Gentiobiose ‡	+	+	L-Arabinose	ND	+	L-Glutamic acid	+	ND
Sucrose ‡	+	+	L-Fructose	-	ND	D-Gluconic acid	+	ND
Stachyose	+	ND	D-Sorbitol*	-	+	D-Glucoronic acid	-	ND
α-D-Lactose	-	+	D-Mannitol ‡	+	+	Glucuronamide	-	ND
D-Salicin ‡	+	+	Glycerol ‡	+	+	P-Hydroxy-Phenylacetic acid	-	ND
N-Acetyl-D-Galactosamine	-	ND	Adonitol	ND	-	Methyl pyruvate	+	ND
α-D-Glucose ‡	+	+	Sorbose	ND	-	L-Lactic acid	+	ND
D-Fructose ‡	+	+	Rhamnose	ND	-	Citric acid	+	ND
3-Methyl glucose	-	ND	Dulcitol	ND	-	D-Malic acid	-	ND
Ribose	ND	+	Inositol	ND	-	L-Malic acid	+	ND
D-xylose	ND	+	α-methyl-D-mannoside	ND	-	α -Hydroxy-Butyric acid	-	ND
L-xylose	ND	-	Melibiose	ND	-	ß-Hydroxy-D,L-Butyric acid	-	ND
Galactose	ND	+	Xylitol	ND	-	α-Keto-Butyric acid	-	ND
Mannose	ND	+	D-Lyxose	ND	-	Acetoacetic acid	+	ND
α-Methyl-D-glucoside	ND	+	D-Tagatose	ND	-	Propionic acid	-	ND
Amygdalin	ND	+	D-Fucose	ND	-	Acetic acid	+	ND
Arbutine	ND	+	D-Arabitol	ND	-	Formic acid	+	ND
Esculin	ND	+	L-Arabitol	ND	-	1% Sodium lactate	+	ND
Inulin	ND	+	Glucanate	ND	-			
Melezitose	ND	+	2-Keto-glucanate	ND	-			
D-raffinose	ND	+	5-Keto-glucanate	ND	-			

(+) Indicate positive result, (-) indicate negative results, (ND) indicate not determined. Wells that showed borderline values are not reported on this table. ‡ Results consistent with those reported by Villegas-Escobar et al. (2013) using API 50 CHB/E system. *Sorbitol reaction was reported as positive using API 50 CHB/E system. Biolog results indicate that strain EA-CB0015 corresponds to *Bacillus safensis*/*pumilus* with a probability of 0.542.

Table S8. Hydrolytic enzymes in *B. subtilis* 168 and *B. tequilensis* EA-CB0015

Gene*	Function	Locus tag 168 (NC_000964.3)	Locus tag EA-CB0015 (NZ_CP048852.1)	Identity %**	Query coverage	E value
ntdA	pyridoxal phosphate-dependent 3-oxo-glucose-6-phosphate:glutamate aminotransferase/ synthesis of the antibiotic kanosamine	BSU_10550	G4P54_RS05605	92.70%	100%	0
ntdB	kanosamine-6-phosphate phosphatase	BSU_10540	G4P54_RS05610	91.58%	99%	0
ntdC	NAD-dependent glucose-6-phosphate dehydrogenase	BSU_10530	G4P54_RS05600	92.98%	100%	0
bglC	endo-1,4-beta-glucanase	BSU_18130	G4P54_RS10355	91.93%	100%	0
bglS	endo-beta-1,3-1,4 glucanase	BSU_39070	G4P54_RS02875	90.96%	98%	0
csn	chitosanase/ chitin degradation	BSU_26890	G4P54_RS13795	89.61%	100%	0
levB	endolevanase/levan degradation	BSU_34460	G4P54_RS17750	90.78%	100%	0
pel	pectate lyase C/ degradation of polygalacturonic acid	BSU_07560	G4P54_RS03980	89.63%	100%	0
pelB	pectate lyase/ degradation of polygalacturonic acid	BSU_18650	G4P54_RS10740	89.70%	100%	0
aprE	extracellular alkaline serine protease (subtilisin E)/ protein degradation	BSU_10300	G4P54_RS05480	93.80%	100%	0
Bpr	bacillopeptidase F/protein degradation	BSU_15300	G4P54_RS08030	89.41%	100%	0
nprE	extracellular neutral protease B	BSU_14700	G4P54_RS07720	93.81%	100%	0
xynC	endo-xylanase, preference for methylglucurono-xylan	BSU_18150	G4P54_RS10395	90.93%	98%	0
xynA	endo-1,4-beta-xylanase/xylan degradation	BSU_18840	G4P54_RS10840	94.39%	100%	0
xynB	xylan beta-1,4-xylosidase/xylan degradation	BSU_17580	G4P54_RS10080	91.32%	100%	0
amyE	alpha-amylase/ starch degradation	BSU_03040	G4P54_RS01735	88.24%	100%	0
amyX	pullulanase (debranching enzyme)	BSU_29930	G4P54_RS15300	87.45%	100%	0
ytnP	lactonase-homolog protein, inhibits the signaling pathway required for the streptomycin production and development of aerial mycelium in Streptomyces griseus/ defense against competing bacteria	BSU_29890	G4P54_RS15280	88.87%	99%	0

yitM	biofilm toxin/ protection of B. subtilis biofilms against competitors	BSU_11040	Absent
yitO	unknown	BSU_11055	Absent
yitP	unknown	BSU_11070	Absent
yizB	similar to transcription regulator (PadR family)	BSU_11079	Absent
yitQ	anti-toxin, confers resistance to YitM	BSU_11080	Absent
yitR	unknown	BSU_11090	Absent
nprB	extracellular neutral protease B, required for the function of the YitM toxin	BSU_11100	Absent

* Genes taken from Subtiwiki

** % identity from blastn

Table S9. Genes associated with phosphate metabolism from *B. subtilis* 168 and B. *tequilensis* EA-CB0015

Gene*	Function	Locus tag 168 (NC_000964.3)	Locus tag EA-CB0015 (NZ_CP048852.1)	Identity %**	Query coverage	E value
glpQ	glycerol-3-phosphate utilization, degradation of wall teichoic acid during phosphate starvation	BSU_02130	G4P54_RS01290	91.39%	98%	0
phoA	aquisition of phosphate upon phosphate starvation	BSU_09410	G4P54_RS05025	89.86%	99%	0
phoB	aquisition of phosphate upon phosphate starvation, ether lipid synthesis	BSU_05740	G4P54_RS03165	92.37%	100%	0
phoD	aquisition of phosphate upon phosphoate starvation, degradation of wall teichoic acid during phosphate starvation	BSU_02620	G4P54_RS01520	92.35%	100%	0
phoP	regulation of phosphate metabolism (*phoA*, *phoB*, *phoD*, *resABCDE*, *tag A-tagB*, *tagDEF*, *tuaA-H*)	BSU_29110	G4P54_RS14870	95.99%	100%	0
phoR	regulation of phosphate metabolism	BSU_29100	G4P54_RS14875	91.26%	100%	0
phy	utilization of inositol hexakisphosphate (phytate)	BSU_19800	G4P54_RS21365 (pseudo gen)	87.11%	19%	2,00E-67
pit	phosphate uptake	BSU_12840	G4P54_RS06790	91.12%	100%	0
ppaC	recovery of phosphate ions from pyrophosphate	BSU_40550	G4P54_RS20815	93.01%	98%	0
pstA	high-affinity phosphate uptake	BSU_24970	G4P54_RS13120	92.54%	100%	0
pstBA	high-affinity phosphate uptake	BSU_24960	G4P54_RS13110	90.86%	100%	0
pstBB	high-affinity phosphate uptake	BSU_24950	G4P54_RS13105	91.40%	99%	0
pstC	high-affinity phosphate uptake	BSU_24980	G4P54_RS13120	94.62%	100%	0
pstS	high-affinity phosphate uptake	BSU_24990	G4P54_RS13125	93.91%	100%	0
sapB	unknown	BSU_06650	G4P54_RS03625	94.56%	100%	0
tatAD	TAT protein secretion	BSU_02630	G4P54_RS01525	96.24%	100%	4,00E-97
tatCD	TAT protein secretion	BSU_02640	G4P54_RS01530	93.74%	98%	0
yqeW	phosphate uptake	BSU_25420	G4P54_RS13335	90.69%	100%	0
yurI	extracellular RNA degradation	BSU_32540	G4P54_RS16665	91.12%	100%	0
ydhF	unknown	BSU_05730	G4P54_RS03160	86.22%	100%	0

* Genes taken from Subtiwiki

** % identity from blastn

Locus tag obtained from Genbank database

Table S10. Genes associated with iron metabolism from *B. subtilis* 168 and B. *tequilensis* EA-CB0015

**Acquisition of iron / Other**						
**Gene***	**Function**	**Locus tag 168 (NC_000964.3)**	**Locus tag EA-CB0015 (NZ_CP048852.1)**	**Identity %****	**Query cover**	**E value**
besA/yuiI	iron acquisition/Ferri-bacillibactin esterase	BSU_32010	G4P54_RS16365	91.03%	98%	0
btr	regulation of iron acquisition/transcriptional activator (AraC family), (AraC family DNA-binding domain fused to FeuA-like substrate-binding domain), regulation of the feuA-feuB-feuC-ybbA operon	BSU_01640	G4P54_RS01050	88.95%	99%	0
cypX	biosynthesis of the extracellular iron chelator pulcherrimin	BSU_35060	Absent			
dhbA	biosynthesis of the siderophore bacillibactin/2,3-dihydro-2,3-dihydroxybenzoate dehydrogenase	BSU_32000	G4P54_RS16360	90.33%	100%	0
dhbB	biosynthesis of the siderophore bacillibactin/isochorismatase	BSU_31970	G4P54_RS16345	92.23%	100%	0
dhbC	biosynthesis of the siderophore bacillibactin/isochorismate synthase	BSU_31990	G4P54_RS16355	88.89%	100%	0
dhbE	biosynthesis of the siderophore bacillibactin/2,3-dihydroxybenzoate-AMP ligase (enterobactin synthetase component E)	BSU_31980	G4P54_RS16350	91.24%	100%	0
dhbF	biosynthesis of the siderophore bacillibactin/involved in 2,3-dihydroxybenzoate biosynthesis	BSU_31960	G4P54_RS16340	89.79%	100%	0
dps	iron storage, survival of of stress conditions	BSU_30650	G4P54_RS15580	93.84%	100%	0
fbpA	RNA chaperone	BSU_04536	G4P54_RS02480	100%	96.97%	2e-75
fbpB	RNA chaperone	BSU_04530	G4P54_RS02475	93.89%	100%	8,00E-74
fbpC	New RNA feature, independent transcript	BSU_22036	G4P54_RS11610	95.45%	97%	1e-34
fur	regulation of iron homoeostasis/transcription regulator of iron homoeostasis, sensor of Fe sufficiency	BSU_23520	G4P54_RS12360	98.22%	100%	0
hmoA	degradation of heme/heme monooxygenase	BSU_07150	G4P54_RS03810	93.27%	100%	7,00E-137
hmoB	degradation of heme, acquisition of iron/heme monooxygenase	BSU_10100	G4P54_RS03810	91.42%	100%	0

mrgA	iron storage/iron storage protein, DNA-binding stress protein, forms highly stable, multimeric protein-DNA complexes which protect against oxidative killing	BSU_32990	G4P54_RS16950	94.16%	100%	0
ybbA	iron acquisition from enterobactin /enterobactin esterase, release of iron from enterobactin	BSU_01600	G4P54_RS01030	87.92%	99%	0
yhfQ	iron uptake/iron/ citrate ABC transporter (solute-binding protein)	BSU_10330	G4P54_RS05495	90.95%	100%	0
ylaN	unknown	BSU_14840	G4P54_RS07800	96.81%	100%	1,00E-133
ymfD/bcbE	iron acquisition/exporter for the siderophore bacillibactin	BSU_16825	G4P54_RS08800	91.10%	97%	0
ytpQ	maybe involved in iron homeostasis	BSU_29830	G4P54_RS15240	94.44%	100%	0
yvmC	biosynthesis of the extracellular iron chelator pulcherrimin	BSU_35070	Absent			
**Acquisition of iron/ based on similarity**						
ytoA	unknown	BSU_30520	G4P54_RS15520	93.41%	100%	0
**ABC transporters for the uptake of iron/ siderophores**						
fecC	iron uptake/iron/ citrate ABC transporter (binding protein)	BSU_07520	G4P54_RS03965	93.14%	100%	0
fecD	acquisition of iron/iron/citrate ABC transporter (permease)	BSU_07510	G4P54_RS03955	92.81%	100%	0
fecE	iron uptake/iron/citrate ABC transporter (permease)	BSU_07500	G4P54_RS03955	93.51%	100%	0
fecF	iron uptake	BSU_07490	G4P54_RS03950	94.76%	100%	0
feuA	acquisition of iron/ABC transporter for the siderophores Fe-enterobactin and Fe-bacillibactin (binding protein), with YusV as ATPase	BSU_01630	G4P54_RS01045	92.24%	100%	0
feuB	acquisition of iron/ABC transporter for the siderophores Fe-enterobactin and Fe-bacillibactin (integral membrane protein)	BSU_01620	G4P54_RS01040	93.43%	100%	0
feuC	acquisition of iron/ABC transporter for the siderophores Fe-enterobactin and Fe-bacillibactin (integral membrane protein)	BSU_01610	G4P54_RS01035	91.49%	100%	0
fhuB	siderophore uptake/hydroxamate siderophore ABC transporter (ferrichrome und ferrioxamine) (permease)	BSU_33310	G4P54_RS17130	88.65%	99%	0
fhuC	siderophore uptake/hydroxamate siderophore ABC transporter (ATP-binding protein) (ferrichrome und ferrioxamine)	BSU_33290	G4P54_RS17125	92.31%	99%	0

fhuD	siderophore uptake/hydroxamate siderophore ABC transporter (only ferrichrome) (binding protein)	BSU_33320	G4P54_RS17140	92.09%	100%	0
fhuG	siderophore uptake/hydroxamate siderophore ABC transporter (ferrichrome und ferrioxamine) (permease)	BSU_33300	G4P54_RS17130	91.20%	100%	0
fpbN	acquisition of iron/petrobactin (3.4-catecholate siderophore) ABC transporter (permease)	BSU_03800	G4P54_RS02100	94.43%	100%	0
fpbO	acquisition of iron/petrobactin (3.4-catecholate siderophore) ABC transporter (permease)	BSU_03810	G4P54_RS02095	95.04%	100%	0
fpbP	acquisition of iron/petrobactin (3.4-catecholate siderophore) ABC transporter (ATP-binding protein)	BSU_03820	G4P54_RS02100	92.75%	100%	0
fpbQ	acquisition of iron/petrobactin (3.4-catecholate siderophore) ABC transporter (binding protein), major component of the secretome	BSU_03830	G4P54_RS02110	95.81%	100%	0
yfhA	acquisition of iron/ABC transporter for the siderophore schizokinen and arthrobactin (permease), works with ATPase YusV	BSU_08460	G4P54_RS04435	90.87%	99%	0
yfiY	acquisition of iron/[SW|ABC transporter for the siderophore schizokinen and arthrobactin (binding protein), works with ATPase YusV	BSU_08440	G4P54_RS04430	93.15%	100%	0
yfiZ	acquisition of iron/ ABC transporter for the siderophore schizokinen and arthrobactin (permease), works with ATPase YusV	BSU_08450	G4P54_RS04435	90.42%	100%	0
yhfQ	iron uptake/iron/ citrate ABC transporter (solute-binding protein)	BSU_10330	G4P54_RS05495	90.95%	100%	0
yusV	acquisition of iron/ABC transporter for the siderophores Fe-enterobactin and Fe-bacillibactin, as well as for the siderophores schizokinen and arthrobactin (ATPase)	BSU_32940	G4P54_RS16930	94.57%	100%	0
yxeB	siderophore uptake/hydroxamate siderophore ABC transporter (only ferrioxamine) (binding protein)	BSU_39610	G4P54_RS20425	90.57%	99%	0
**Elemental iron transport system**						
efeB	ferrous iron conversion/elemental iron uptake system, heme peroxidase, converts ferrous iron (Fe(II) to ferric iron (FeIII)) for uptake by EfeO-EfeU, peroxide detoxification under microaerobic conditions	BSU_38260	G4P54_RS19740	91.85%	100%	0
efeO	elemental iron uptake/lipoprotein, elemental iron uptake system (binding protein), high affinity uptake of ferric iron (Fe(III))	BSU_38270	G4P54_RS19745	92.14%	100%	0

efeU	elemental iron uptake/elemental iron uptake system (permease), high affinity uptake of ferric iron (Fe(III))	BSU_38280	G4P54_RS19745	91.36%	100%	0
**Iron export**						
pfeT	protection against toxic iron/Fe2+ efflux pump, P1B4-type ATPase, protects the cell against iron intoxication	BSU_13850	G4P54_RS07285	92.22%	100%	0
**Biosynthesis of iron-sulfur clusters**						
fra	intracellular iron channeling, biosynthesis of iron-sulfur clusters	BSU_05750	Absent			
sufA	assembly of Fe-S clusters	BSU_32160	G4P54_RS16435	94.48%	99%	1,00E-159
sufB	synthesis of iron-sulfur clusters	BSU_32670	G4P54_RS16785	97.07%	100%	0
sufC	synthesis of Fe-S clusters/ABC transporter (ATP-binding protein), synthesis of Fe-S clusters	BSU_32710	G4P54_RS16805	96.69%	100%	0
sufD	synthesis of Fe-S-clusters/FeS scaffold, synthesis of Fe-S-clusters	BSU_32700	G4P54_RS16800	96.65%	100%	0
sufS	formation of iron-sulfur clusters in proteins/cysteine desulfurase, cysteine:SufU sulfuryl transferase	BSU_32690	G4P54_RS16795	96.72%	100%	0
sufU	transfer of sulfur from SufS to SufB/sulfur shuttle protein, receives sulfur from SufS, transfers it to SufB	BSU_32680	G4P54_RS16790	97.07%	100%	0
yitW	assembly of iron-sulphur clusters/iron-sulphur cluster assembly factor	BSU_11160	G4P54_RS05890	92.56%	100%	3,00E-125

* Genes taken from Subtiwiki

** % identity from Blastn

Locus tag obtained from Genbank database

Table S11. Genes associated with UV and oxidative stress response

Gene*	Function	Locus tag 168 (NC_000964.3)	Locus tag EA-CB0015 (NZ_CP048852.1)	Identity %**	Query cover	E value
**Photoreceptors**						
ytvA	blue light sensor, positive regulation of SigB activity under conditions of blue light	BSU_30340	G4P54_RS15420	88.42%	100%	0
**Photolyases**						
splB	spore photoproduct lyase, radical SAM enzyme	BSU_13930	G4P54_RS07330	92.61%	100%	0
splA	transcriptional repressor of the spore photoproduct lyase splA-splB operon	BSU_13920	G4P54_RS07325	90.83%	100%	1,00E-88
**Enzymatic antioxidants**						
katA	main vegetative catalase 1/detoxification (degradation) of hydrogen peroxide	BSU_08820	G4P54_RS04715	93.80%	100%	0
katE	catalase, general stress protein/detoxification (degradation) of hydrogen peroxide	BSU_39050	G4P54_RS20130	91.36%	100%	0
katX	catalase, general stress protein/detoxification (degradation) of hydrogen peroxide	BSU_38630	G4P54_RS19935	90.69%	99%	0
trxA	thioredoxin / protection of proteins against oxidative damage	BSU_28500	G4P54_RS14565	97.14%	100%	6e-152
trxB	thioredoxin reductase (NADPH)/ keeps thioredoxin in the reduced state	BSU_34790	G4P54_RS17925	94.01%	100%	0
sodA	superoxide dismutase/ detoxification of oxygen radicals	BSU_25020	G4P54_RS13140	94.91%	100%	0
sodF	superoxide dismutase/ detoxification of oxygen radicals	BSU_19330	G4P54_RS11150	89.36%	100%	0
ahpC	alkyl hydroperoxide reductase (small subunit)/resistance against peroxide stress	BSU_40090	G4P54_RS20595	97.16%	100%	0
ahpF	alkyl hydroperoxide reductase (large subunit) / NADH dehydrogenase/resistance against peroxide stress	BSU_40100	G4P54_RS20600	94.12%	100%	0
ohrA	peroxiredoxin, protects the cell against organic peroxides/ organic peroxide resistance	BSU_13140	G4P54_RS06930	88.73%	100%	2,00E-148
**Non-enzymatic antioxidants**						
bshA	biosynthesis of bacillithiol/ L-malic acid glycosyltransferase, involved in bacillithiol synthesis	BSU_22460	G4P54_RS11815	91.89%	100%	0
bshC	biosynthesis of bacillithiol/cysteine-adding enzyme required for the synthesis of bacillithiol	BSU_15120	G4P54_RS07940	91.98%	100%	0

bshB1	biosynthesis of bacillithiol/ N-acetylglucosamine-malate deacetylase, involved in bacillithiol synthesis (major enzyme)	BSU_22470	G4P54_RS11815	90.58%	100%	0
bshB2	biosynthesis of bacillithiol/ N-acetylglucosamine-malate deacetylase, minor enzyme involved in bacillithiol synthesis, may act as amidase for the processing of BSH conjugates	BSU_19460	G4P54_RS11220	93.99%	100%	0
non-mevalonate pathway or the MEP/DOXP pathway						
	biosynthesis of phytoene/K02291 15-cis-phytoene synthase [EC:2.5.1.32]	Absent	G4P54_RS05740			
sqhC	Sporulene/ squalene-hopene cyclase, biosynthesis of sporulenes, protection of the spore against oxidative stress	BSU_19320	G4P54_RS11150	89.62%	99%	0
**Other Resistance against oxidative and electrophile stress**						
aag	DNA repair, survival of stress conditions	BSU_38620	G4P54_RS19930	93,23%	100%	0
adhA	response to toxic formaldehyde	BSU_27010	Absent			
adhR	regulation of the protective response to formaldehyde and methylglyoxal	BSU_27000	Absent			
ahpT/ykuV	protection of proteins against oxidative damage	BSU_14230	G4P54_RS07475	90,99%	99%	5,18E-172
aldY	stress resistance	BSU_38830	G4P54_RS20030	90,05%	100%	0
azoR1/azoJ	quinone detoxification	BSU_19230	G4P54_RS11100	95,85%	100%	0
azoR2/azoRB	resistance to 2-methylhydroquinone	BSU_33540	G4P54_RS17250	94,18%	100%	0
brxA	de-bacillithiolation of S-bacillithiolated OhrR and MetE	BSU_21860	G4P54_RS11510	94,25%	100%	0
brxB	de-bacillithiolation of S-bacillithiolated OhrR and MetE	BSU_23990	G4P54_RS12605	94,74%	100%	0
bsaA	unknown	BSU_21900	G4P54_RS11530	89,57%	97%	2,02E-171
bstA	detoxification	BSU_08390	G4P54_RS04410	90,31%	100%	0
catD	detoxification of catechol	BSU_08230	G4P54_RS04345	90,37%	100%	4,81E-152
catE	detoxification of catechol	BSU_08240	G4P54_RS04350	89,72%	99%	0
catR	resistance against oxidative and electrophile stress	BSU_33680	G4P54_RS17335	96,02%	100%	3,83E-152
csbA	protection against paraquat stress	BSU_35180	G4P54_RS18115	95,67%	100%	6,10E-104
csbB	lipoteichoic acid glycosylation , survival of stress conditions	BSU_08600	G4P54_RS04510	92,83%	100%	0
csbC	protection against paraquat stress	BSU_39810	G4P54_RS20470	91,56%	100%	0
cypC	biosynthesis of beta-hydroxy fatty acid for lipopeptides	BSU_02100	G4P54_RS01275	89,79%	99%	0

dps	iron storage, survival of of stress conditions	BSU_30650	G4P54_RS15580	93,83%	100%	0
efeB	ferrous iron conversion	BSU_38260	G4P54_RS19740	91,84%	100%	0
glxA	detoxification of methylglyoxal	BSU_38370	G4P54_RS19795	93,70%	100%	9,55E-164
glxB	detoxification of methylglyoxal	BSU_32660	Absent			
guaD	deamination of guanine to xanthine, purine salvage and interconversion	BSU_13170	G4P54_RS06950	89,19%	100%	1,19E-168
hpf	protection of essential ribosomal proteins (S2 and S3)	BSU_35310	G4P54_RS18190	95,96%	100%	0
hxlA	ribulose monophosphate pathway for formaldehyde fixation	BSU_03460	G4P54_RS01950	93,68%	100%	0
hxlB	ribulose monophosphate pathway for formaldehyde fixation	BSU_03450	G4P54_RS01945	90,50%	100%	0
hxlR	regulation of the ribulose monophosphate pathway	BSU_03470	G4P54_RS01955	94,21%	100%	9,13E-159
hypO	protection against NaOCl stress	BSU_07830	G4P54_RS04105	94,14%	100%	0
hypR	control of the nitroreductase gene hypO in response to disulfide stress (diamide, NaOCl)	BSU_40540	G4P54_RS20810	93,91%	100%	9,47E-164
liaF	control of LiaR activity	BSU_33100	G4P54_RS17020	94,35%	100%	0
liaG	unknown	BSU_33110	G4P54_RS17025	86,02%	100%	0
liaH	resistance against oxidative stress and cell wall antibiotics, protein secretion	BSU_33120	G4P54_RS17030	93,09%	100%	0
liaI	resistance against oxidative stress and cell wall antibiotics	BSU_33130	G4P54_RS17035	96,06%	100%	9,35E-179
liaR	regulation of the *liaI-liaH-liaG-liaF-liaS-liaR* operon	BSU_33080	G4P54_RS17010	92,76%	100%	0
liaS	control of LiaR activity in response to bacitracin	BSU_33090	G4P54_RS17015	91,78%	100%	0
mgsR	controls a subset of general stress genes	BSU_24770	G4P54_RS13015	90,90%	98%	3,53E-143
mhqA	resistence to methyl-hydroxyquinone	BSU_12870	G4P54_RS06805	91,90%	100%	0
mhqD	may be involved in protection against methyl-hydroquinone	BSU_19560	G4P54_RS11260	90,54%	100%	0
mhqE	resistance to methyl hydroquinone and catechol	BSU_19570	G4P54_RS11265	90,14%	99%	0
mhqN	unknown	BSU_05480	G4P54_RS03025	89,69%	100%	0
mhqO	resistence to methyl-hydroxyquinone	BSU_05490	G4P54_RS03030	88,90%	99%	0
mhqP	may be involved in protection against methyl-hydroquinone	BSU_05500	G4P54_RS03035	91,28%	100%	4,62E-152
mhqR	regulation of resistance to quinones and diamide	BSU_13670	G4P54_RS07195	95,66%	100%	0
mrgA	iron storage,	BSU_32990	G4P54_RS16950	94,15%	100%	0
msrA	regeneration of methionine and restoration of protein function after oxidative damage	BSU_21690	G4P54_RS11425	95,51%	100%	0

msrB	regeneration of methionine and restoration of protein function after oxidative damage	BSU_21680	G4P54_RS11420	91,95%	100%	2,99E-174
nfrA	unknown	BSU_38110	G4P54_RS19660	91,73%	100%	0
ohrB	general stress protein/organic peroxide resistance	BSU_13160	G4P54_RS06940	92,46%	100%	2,00E-168
ohrR	regulation of ohrA expression in response to organic peroxides	BSU_13150	G4P54_RS06935	90,10%	93%	1,14E-153
perR	regulation of the response to peroxide/ transcriptional repressor of the peroxide regulon, sensor of the intracellular Fe/Mn ratio	BSU_08730	G4P54_RS04575	96,58%	100%	0
rnr	nonspecific degradation of rRNA	BSU_33610	G4P54_RS17300	93,24%	99%	0
spx	negative and positive regulator of many genes	BSU_11500	G4P54_RS06065	96,97%	100%	0
tpx	unknown	BSU_29490	G4P54_RS15070	96,82%	100%	0
tsaE	control of tRNA modification	BSU_05910	G4P54_RS03275	93,71%	100%	0
ydaG	survival of stress conditions	BSU_04220	G4P54_RS02315	92,91%	100%	4,86E-177
ydeA/sufLC	detoxification of methylglyoxal	BSU_05110	Absent			
ydhK	survival of stress conditions	BSU_05790	G4P54_RS03180	89,08%	95%	0
yerD	protection against paraquat stress	BSU_06590	G4P54_RS03595	89,29%	100%	0
yfhE	survival of stress conditions and low temperatures	BSU_08500	Absent			
yfkH	survival of stress conditions	BSU_07900	G4P54_RS04140	92,87%	100%	0
yfkI	protection against paraquat stress	BSU_07890	G4P54_RS04135	92,84%	100%	3,86E-132
yfkJ	survival of stress conditions	BSU_07880	Absent			
yfkM	detoxification of methylglyoxal	BSU_07850	G4P54_RS04115	90,77%	100%	0
yfkS	germination, survival of of stress conditions	BSU_07770	G4P54_RS04075	92,46%	99%	7,01E-78
yflA	protection against paraquat stress	BSU_07750	G4P54_RS04065	88,98%	100%	0
yhdA	protection against oxidative stress	BSU_09340	G4P54_RS04990	81,59%	89%	2,44E-96
yhdN	detoxification of methylglyoxal	BSU_09530	G4P54_RS05085	89,86%	100%	0
yitT	protection against paraquat stress	BSU_11120	G4P54_RS05860	94,54%	100%	0
yjbC	survival of paraquat stress	BSU_11490	G4P54_RS06060	96,54%	100%	0
yjgD	survival of stress conditions	BSU_12170	G4P54_RS06460	92,51%	100%	0
ylxP	protection against paraquat stress	BSU_16640	Absent			
ymaD	protection against oxidative stress	BSU_17280	G4P54_RS08960	91,83%	100%	0

yodB	regulation of quinone and diamide detoxification	BSU_19540	G4P54_RS11190	90,88%	100%	0
yodC	regeneration of NAD from NADH	BSU_19550	G4P54_RS11250	92,31%	99%	1,46E-136
yojM	unknown	BSU_19400	G4P54_RS11255	94,25%	100%	0
ypdA	recycling of oxidized bacillithiol disulfide to the reduced form (BSH)	BSU_22950	G4P54_RS12070	91,43%	99%	0
yqhB	protection against stress conditions	BSU_24750	G4P54_RS12510	93,02%	100%	0
yqjL	resistence against paraquat	BSU_23830	G4P54_RS12515	91,86%	100%	0
yqjM	reduction of double bonds of nonsaturated aldehydes	BSU_23820	G4P54_RS13000	90,97%	100%	0
yraA	detoxification of methylglyoxal	BSU_27020	G4P54_RS13810	93,53%	100%	0
ytpQ	maybe involved in iron homeostasis	BSU_29830	G4P54_RS15240	94,44%	100%	0
yugU	survival of stress conditions	BSU_31280	G4P54_RS16000	92,13%	99%	2,81E-159
yvgN	unknown	BSU_33400	G4P54_RS17180	90,63%	100%	0
ywiE	phospholipiid biosynthesis, protection against paraquat stress	BSU_37240	G4P54_RS19240	91,68%	100%	0
yxiS	survival of stress conditions	BSU_39040	G4P54_RS21430	95,06%	99%	1,15E-116
yxjJ	survival of stress conditions	BSU_38930	G4P54_RS20125	93,50%	95%	4,59E-116

* Genes taken from Subtiwiki

** % identity from blastn

Locus tag obtained from Genbank database

Table S12. Natural product biosynthetic gene clusters present in the genome of *B. tequiliensis* EA-CB0015

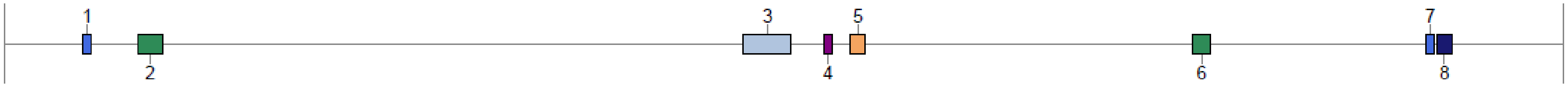

Region*	Type	From	To	Most similar known cluster		Similarity
Region 1	Ranthipeptide,sactipeptide	200,026	222,619	Sporulation killing factor	RiPP:Head-to-tailcyclized peptide	100%
Region 2	NRPS	340,165	404,921	Surfactin	NRP:Lipopeptide	69%
Region 3	NRPS,transAT-PKS,betalactone	1,900,134	2,023,672	Fengycin	NRP	100%
Region 4	Terpene	2,107,226	2,129,124			
Region 5	T3PKS	2,174,022	2,215,119			
Region 6	NRPS	3,055,813	3,102,967	Bacillibactin	NRP	100%
Region 7	Sactipeptide	3,657,131	3,678,742	Subtilosin A	RiPP:Thiopeptide	100%
Region 8	Other	3,684,092	3,725,510	Bacilysin	Other	100%
Region 9**	Saccharide	3,801,489	3,831,329	Iturin	Polyketide + NRP:Lipopeptide	40%

*Regions predicted with antiSMASH version 6.1.1 in relaxed mode.
** Region 9 was predicted with antiSMASH version 6.1.1 in loose mode

Table S13. Annotation of putative terpene BGC neighborhood in *B. tequilensis* EA-CB0015

#	Locus tag (NZ_CP048852.1)	From	To	strand	CDS Annotation	Length (AA)	Pfam Family Domain Id	Accession number	Description Pfam Annotation	Bit score	E-value
**1**	G4P54_RS 11085	2107966	2108829	-	LysM peptidoglycan-dinding domain-containing protein	288	LysM	PF01476.20	LysM domain	52.57	3.50E-14
							LysM	PF01476.20	LysM domain	52.52	3.70E-14
							3D	PF06725.11	3D domain	78.37	3.50E-22
**2**	G4P54_RS 11090	2109076	2110851	-	DNA helicase RecQ	592	DEAD	PF00270.29	DEAD/DEAH box helicase	62.3	4.70E-17
							Helicase_C	PF00271.31	Helicase conserved C-terminal domain	59.88	2.70E-16
							RecQ_Zn_bind	PF16124.5	RecQ zinc-binding	62.92	3.40E-17
							RQC	PF09382.10	RQC domain	100.61	4.30E-29
							HRDC	PF00570.23	HRDC domain	80.36	7.20E-23
**3**	G4P54_RS 11100	2111413	2112039	-	FMN-dependent NADH-azoreductase	209	Flavodoxin_2	PF02525.17	Flavodoxin-like fold	151.93	1.70E-44
**4**	G4P54_RS 11105	2112190	2112681	-	TraR/DksA family transcriptional regulator	164	zf-dskA_traR	PF01258.17	Prokaryotic dksA/traR C4-type zinc finger	38.15	1.00E-09
**5**	G4P54_RS 11110	2112756	2113088	-	hypothetical protein	111	NA	NA	No hits found for query	NA	NA
**6**	G4P54_RS 11115	2113165	2113392	+	hypothetical protein	76	NA	NA	No hits found for query	NA	NA
**7**	G4P54_RS 11120	2113379	2113855	-	Hsp20/alpha crystallin family protein	159	HSP20	PF00011.21	Hsp20/alpha crystallin family	35.81	6.70E-09
**8**	G4P54_RS 11125	2113922	2114182	+	hypothetical protein	87	HSP20	PF00011.21	Hsp20/alpha crystallin family	35.81	6.70E-09
**9**	G4P54_RS 11130	2114187	2114420	+	hypothetical protein	78	NA	NA	No hits found for query	NA	NA
**10**	G4P54_RS 11135	2114501	2114845	-	hypothetical protein	115	bPH_1	PF08000.11	Bacterial PH domain	86.04	1.90E-24
**11**	G4P54_RS 11140	2115209	2115412	-	hypothetical protein	68	NA	NA	No hits found for query	NA	NA
**12**	G4P54_RS 11145	2115642	2117129	+	aldehyde dehydrogenase DhaS	496	Aldedh	PF00171.22	Aldehyde dehydrogenase family	633.44	1.70E-190
13	G4P54_RS 11150	2117226	2119124	+	squalene--hopene cyclase	633	SQHop_cyclase_N	PF13249.6	Squalene-hopene cyclase N-terminal domain	181.71	1.60E-53
							SQHop_cyclase_C	PF13243.6	Squalene-hopene cyclase C-terminal domain	303.8	1.30E-90

**14**	G4P54_RS 11155	2119114	2119959	+	superoxide dismutase	282	Sod_Fe_N	PF00081.22	Iron/manganese superoxide dismutases, alpha-hairpin domain	91.95	2.50E-26
							Sod_Fe_C	PF02777.18	Iron/manganese superoxide dismutases, C-terminal domain	115.06	1.40E-33
**15**	G4P54_RS 11160	2119993	2121330	-	sodium-dependent transporter	446	SNF	PF00209.18	Sodium:neurotransmitter symporter family	91.76	4.10E-26
							SNF	PF00209.18	Sodium:neurotransmitter symporter family	97.37	8.20E-28
**16**	G4P54_RS 11165	2121559	2122524	+	bile acid:sodium symporter family protein	322	SBF	PF01758.16	Sodium Bile acid symporter family	177.08	3.10E-52
**17**	G4P54_RS 11170	2122573	2123817	-	2-oxoglutarate dehydrogenase complex dihydrolipoyllysine-residue succinyltransferase	415	Biotin_lipoyl	PF00364.22	Biotin-requiring enzyme	81.31	3.30E-23
							E3_binding	PF02817.17	e3 binding domain	46.68	3.00E-12
							2-oxoacid_dh	PF00198.23	2-oxoacid dehydrogenases acyltransferase (catalytic domain)	265.01	5.10E-79
**18**	G4P54_RS 11175	2123833	2126667	-	2-oxoglutarate dehydrogenase E1 component	945	2-oxogl_dehyd_N	PF16078.5	2-oxoglutarate dehydrogenase N-terminus	28.43	8.80E-07
							E1_dh	PF00676.20	Dehydrogenase E1 component	330.78	6.00E-99
							Transket_pyr	PF02779.24	Transketolase, pyrimidine binding domain	195.87	4.40E-58
							OxoGdeHyase_C	PF16870.5	2-oxoglutarate dehydrogenase C-terminal	132.99	6.80E-39
**19**	G4P54_RS 11180	2126896	2128812	-	nitric oxide reductase activation protein NorD	639	VWA	PF00092.28	von Willebrand factor type A domain	34.24	2.70E-08

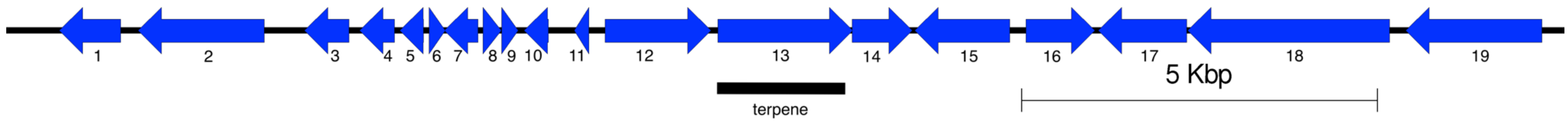

Table S14. Annotation of putative type III PKS BGC neighborhood in *B. tequilensis* EA-CB0015

#	Locus tag (NZ_CP048852.1)	From	To	strand	CDS Annotation	Length (AA)	Pfam Family Domain Id	Accession number	Description Pfam Annotation	Bit score	E-value
**1**	G4P54_RS11490	2174211	2175005	-	thymidylate synthase	265	Thymidylat_synt	PF00303.19	Thymidylate synthase	425.06	8.80E-128
**2**	G4P54_RS11495	2175089	2175622	-	phosphatidylglycerophosphatase A	178	PgpA	PF04608.13	Phosphatidyl glycerophosphatase A	68.81	4.80E-19
**3**	G4P54_RS11500	2175640	2176254	-	hypothetical protein	205	YpjP	PF14005.6	YpjP-like protein	201.23	5.60E-60
**4**	G4P54_RS11505	2176518	2177303	-	class I SAM-dependent methyltransferase	262	SAM_MT	PF04445.13	Putative SAM-dependent methyltransferase	59.34	3.50E-16
**5**	G4P54_RS11510	2177345	2177779	-	bacilliredoxin BrxA	145	Disulph_isomer	PF06491.11	Disulphide isomerase	212.29	2.40E-63
**6**	G4P54_RS11515	2177886	2179562	-	dihydroxy-acid dehydratase	559	ILVD_EDD	PF00920.21	Dehydratase family	764.15	6.40E-230
**7**	G4P54_RS11520	2179853	2180986	-	virulence factor	378	Virulence_fact	PF13769.6	Virulence factor	104.65	2.30E-30
**8**	G4P54_RS11525	2181046	2181663	-	HD domain-containing protein	206	Thymidylat_synt	PF00303.19	Thymidylate synthase	425.06	8.80E-128
**9**	G4P54_RS11530	2181677	2182159	-	glutathione peroxidase	161	GSHPx	PF00255.19	Glutathione peroxidase	139.77	2.20E-41
**10**	G4P54_RS11535	2182450	2183355	+	homoserine O-succinyltransferase	302	HTS	PF04204.16	Homoserine O-succinyltransferase	457.77	1.20E-137
**11**	G4P54_RS11540	2183582	2184730	+	diglucosyl diacylglycerol synthase	383	MGDG_synth	PF06925.11	Monogalactosyldiacylglycerol (MGDG) synthase	161.32	2.20E-47
**12**	G4P54_RS11545	2184787	2184954	+	Hypothetical protein		Glycos_transf_1	PF00534.20	Glycosyl transferases group 1	71.52	5.80E-20
**13**	G4P54_RS11550	2184970	2185170	+	cold-shock protein	67	CSD	PF00313.22	Cold-shock' DNA-binding domain	101.56	1.60E-29
**14**	G4P54_RS11555	2185222	2185404	-	transcriptional regulator DegR	61	NA	NA	No hits found for query	NA	NA
**15**	G4P54_RS11560	2185560	2185829	+	DUF2564 family protein	90	DUF2564	PF10819.8	Protein of unknown function (DUF2564)	98.39	2.30E-28
**16**	G4P54_RS11565	2185858	2186040	-	zinc-finger domain-containing protein	61	zf-C2HCIx2C	PF10782.9	Zinc-finger	100.07	4.80E-29
**17**	G4P54_RS11570	2186033	2186713	-	ribonuclease H family protein	254	RVT_3	PF13456.6	Reverse transcriptase-like	52.75	3.30E-14

**18**	G4P54_RS11575	2186796	2187485	+	queuosine precursor transporter	230	Vut_1	PF02592.15	Putative vitamin uptake transporter	156	8.50E-46
**19**	G4P54_RS11575	2187485	2187883	+	reverse transcriptase-like protein	133	RVT_3	PF13456.6	Reverse transcriptase-like	66.06	2.50E-18
**20**	G4P54_RS11585	2187923	2188051	+	small, acid-soluble spore protein L	43	NA	NA	No hits found for query	NA	NA
**21**	G4P54_RS11590	2188060	2188950	-	5'-3' exonuclease	297	5_3_exonuc_N	PF02739.16	5'-3' exonuclease, N-terminal resolvase-like domain	165.21	1.00E-48
**22**	G4P54_RS11595	2189051	2189197	-	hypothetical protein	49	NA	NA	No hits found for query	NA	NA
**23**	G4P54_RS11600	2189271	2189528	-	YpbS family protein	86	DUF2533	PF10752.9	Protein of unknown function (DUF2533)	136.29	3.60E-40
**24**	G4P54_RS11605	2189595	2193176	-	dynamin family protein	1194	Dynamin_N	PF00350.23	Dynamin family	83.87	1.30E-23
25	G4P54_RS11615 (*gpsB*)	2193511	2194018	-	isoprenylcysteine carboxyl methyltransferase family protein	169	ICMT	PF04140.14	Isoprenylcysteine carboxyl methyltransferase (ICMT) family	100.37	5.80E-29
26	G4P54_RS11620 (*gpsA*)	2194021	2195119	-	type III polyketide synthase	366	Chal_sti_synt_N	PF00195.19	Chalcone and stilbene synthases, N-terminal domain	68.08	7.30E-19
							Chal_sti_synt_C	PF02797.15	Chalcone and stilbene synthases, C-terminal domain	66.88	1.90E-18
**27**	G4P54_RS11625	2195193	2196506	-	xanthine permease	438	Xan_ur_permease	PF00860.20	Permease family	417.75	3.50E-125
**28**	G4P54_RS11630	2196503	2197087	-	xanthine phosphoribosyltransferase	195	Pribosyltran	PF00156.27	Phosphoribosyl transferase domain	31.69	9.60E-08
**29**	G4P54_RS11635	2197419	2198924	-	carboxypeptidase	502	Peptidase_M32	PF02074.15	Carboxypeptidase Taq (M32) metallopeptidase	640.54	1.70E-192
**30**	ctg1_2238	2199013	2199214	+	hypothetical protein	67	NA	NA	No hits found for query	NA	NA
**31**	G4P54_RS11645	2199610	2201535	-	ATP-dependent DNA helicase	642	Helicase_C_2	PF13307.6	Helicase C-terminal domain	87.72	9.60E-25
**32**	G4P54_RS11650	2201639	2201830	-	hypothetical protein	64	NA	NA	No hits found for query	NA	NA
**33**	G4P54_RS11655	2201993	2202145	+	YpzG family protein	51	YpzG	PF14139.6	YpzG-like protein	90.1	7.20E-26
**34**	G4P54_RS11660	2202197	2203354	-	class I SAM-dependent RNA methyltransferase	386	THUMP	PF02926.17	THUMP domain	70.87	1.20E-19
**35**	G4P54_RS11670	2203903	2204202	-	DUF1273 family protein	100	DivIVA	PF05103.13	DivIVA protein	57.86	1.10E-15
**36**	G4P54_RS11675	2204279	2204821	-	DUF1273 domain-containing protein	181	YpsA	PF06908.11	YspA SLOG family	237.85	6.00E-71

**37**	G4P54_RS11680	2204910	2205131	-	spore coat protein	74	Spore-coat_CotD	PF11122.8	Inner spore coat protein D	60.49	2.20E-16
**38**	G4P54_RS11685	2205214	2205309	-	hypothetical protein	32	NA	NA	No hits found for query	NA	NA
**39**	G4P54_RS11690	2205443	2206693	-	ribonuclease H-like domain-containing protein	417	RNase_H_2	PF13482.6	RNase_H superfamily	127.91	3.60E-37
**40**	G4P54_RS11695	2206709	2208958	-	DEAD/DEAH box helicase	750	DEAD	PF00270.29	DEAD/DEAH box helicase	97.65	6.60E-28
**41**	G4P54_RS11700	2209061	2209591	-	PTS system transporter subunit IIA	177	PTS_EIIA_1	PF00358.20	phosphoenolpyruvate-dependent sugar phosphotransferase system, EIIA 1	177.13	1.10E-52
**42**	G4P54_RS11705	2209705	2210124	+	hypothetical protein	140	NA	NA	No hits found for query	NA	NA
**43**	G4P54_RS11710	2210145	2210516	-	hypothetical protein	124	YppG	PF14179.6	YppG-like protein	100.7	6.60E-29
**44**	G4P54_RS11715	2210703	2210891	+	hypothetical protein	63	YppF	PF14178.6	YppF-like protein	94.43	2.60E-27
**45**	G4P54_RS11720	2210931	2211302	-	YppE family protein	124	DUF1798	PF08807.10	Bacterial domain of unknown function (DUF1798)	118.96	9.80E-35
**46**	G4P54_RS11725	2211335	2211580	-	YppD family protein	82	DUF5446	PF17522.2	Family of unknown function (DUF5446)	108.18	1.70E-31
**47**	G4P54_RS11730	2211778	2211882	+	acid-soluble spore protein SspM	35	NA	NA	No hits found for query	NA	NA
**48**	G4P54_RS11735	2211907	2212869	-	DUF2515 domain-containing protein	321	DUF2515	PF10720.9	Protein of unknown function (DUF2515)	431.06	2.30E-129
**49**	G4P54_RS11740	2212910	2213530	+	Holliday junction resolvase RecU	207	RecU	PF03838.14	Recombination protein U	216.75	1.60E-64

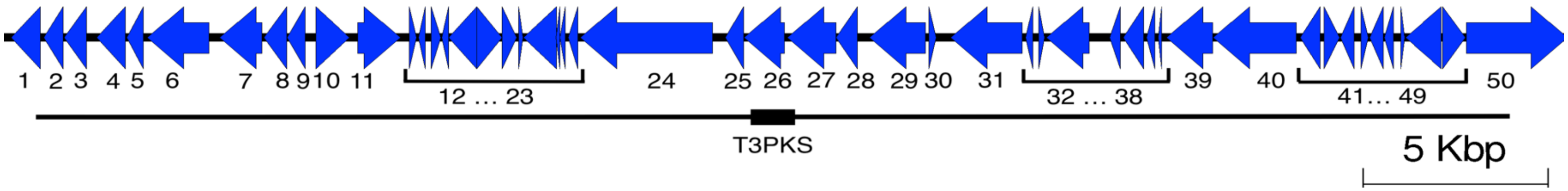

Table S15. Indole-3 acetic acid (IAA) pathways identify in *B. tequilensis* EA-CB0015

**Pathway**	**Enzymes proposed in each step**	**Putative genes in EA-CB0015 (locus_tag*)**	**Reference**
IAM	Tryptophan 2 mono-oxygenase	Not detected	KEGG bteq 00380
	Indol acetamide hydrolase	Not detected	KEGG bteq 00380
IPyA	Tryptophan (amino) transferase	*patB* (lG4P54_RS16090)	Shao et al. 2015
	Tryptophan side chain oxidase	Not detected	KEGG bteq 00380
	Indole-3-pyruvate decarboxylase	*yclC/bsdC* (G4P54_RS02025)	Shao et al. 2015
	Indole 3-acetaldehyde dehydrogenase	*dhaS* (G4P54_RS11145)	KEGG bteq 00380
TAM	Trp decarboxylase	*bsdC/yclC* (G4P54_RS02025)	Shao et al. 2015
	Monoamine oxidase	flavin monoamine oxidase family protein (G4P54_10790)	KEGG bteq 00380
	IAAld dehydrogenase	*dhaS* (G4P54_RS11145)	Shao et al. 2015
IAN	Nitrilase	*yhcX* (G4P54_RS04945)	Shao et al. 2015
other	*ysnE*	Not detected	Shao et al. 2015

IAM = inole 3-acetamide

IPyA = indole 3-pyruvic acid

TAM = trypthamine

IAN = indole 3-acetonitrile pathway

*Locus tag obtained from Genbank database

Table S16. Genes associated with the integrative or conjugative element (ICEBs1) in *B. tequilensis* EA-CB0015

**Conjugation ICEBs1**		**Locus tag 168 (NC_000964.3)**	**Locus tag EA-CB0015 (NZ_CP048852.1)**	**Identity %****	**Query cover**	**E value**
**Name**	**Function**					
conB	conjugative transfer of ICEBs1	BSU_04910	Absent			
conC	conjugative transfer of ICEBs1	BSU_04920	Absent			
conD	conjugative transfer of ICEBs1	BSU_04930	Absent			
conE	conjugative transfer of ICEBs1	BSU_04940	Absent			
conG	conjugative transfer of ICEBs1	BSU_04960	Absent			
cwlT	conjugative transfer of ICEBs1	BSU_04970	G4P54_RS02660 Pseudogene	94.85%	76%	2,00E-162
immA	control of ImmR activity/ protease, degrades ImmR at a specific site	BSU_04810	G4P54_RS02650 Pseudogene	99.71%	66%	2,00E-178
immR	control of transfer of the mobile genetic element ICEBs1	BSU_04820	Absent			
int	excision of the conjugative transposon ICEBs1 from the trnS-leu2 locus/integrase	BSU_04800	G4P54_RS02645	99.10%	100%	0
	hypothetical protein/site specific integrase (blastP against other bacillus)	Absent	G4P54_RS02655			
nicK	conjugation of ICE BS1/DNA relaxase, similar to transposon protein	BSU_04870	Absent			
phrI	control of the transfer of the mobile genetic element ICEBs1/response regulator aspartate phosphatase (RapI) regulator	BSU_05020	G4P54_RS02670 Pseudogene	92.37%	98%	7e-43
rapI	control of transfer of the mobile genetic element ICEBs1/response regulator aspartate phosphatase, antagonist of ImmR	BSU_05010	Absent			
xis	excision of the conjugative transposon ICEBs1 from the trnS-leu2 locus/excisionase	BSU_04830	Absent			
ydcO	unknown	BSU_04840	Absent			
ydcP	conjugation and replication of ICEBs1	BSU_04850	Absent			
ydcQ	conjugative transfer of ICEBs1	BSU_04860	Absent			
ydcS	unknown	BSU_04880	Absent			
ydcT	unknown	BSU_04890	Absent			
yddA	unknown	BSU_04900	Absent			
yddF	unknown	BSU_04950	Absent			
yddI	unknown	BSU_04980	Absent			
yddJ	prevention of redundant transfer of ICEBs1 into host cells that already contain a copy of the element	BSU_04990	Absent			

yddK	unknown	BSU_05000	Absent			
yddM	unknown	BSU_05030	Absent			
ydzL	unknown	BSU_04839	Absent			
**Additional genes**						
pcrA	ATP-dependent DNA helicase, coupling of DNA replication, transcription, recombination and segregation, facilitates unwinding of ICEBs1 DNA for horizontal transfer	BSU_06610	G4P54_RS03605	93.83%	100%	0
yerB	PcrA interaction protein	BSU_06570	G4P54_RS03585	94.48%	100%	0

* Genes taken from Subtiwiki

** % identity from blastn

Locus tag obtained from Genbank database

Table S17. Insertion sequences (ISs) in the genome of *B. tequilensis* EA-CB0015

IS Family	Group	IS	Size (bp)	DR	Start	Stop	Alignment (bp)
IS1182	-	ISBspe1	1560	AGCAGCTGAATTATTCAGCTGCT	804412	804467	55 (Pseudogene)
IS1595	ISPna2	ISPana1*	2006	AAAACAAA	3060338	3060619	281 (Pseudogene)
		ISBsu3*	1723	AATTTATA	3060141	3060319	178 (Pseudogene)
		ISBwe1*	1536	-	3060421	3060546	125 (Pseudogene)
		ISBko1‡	1721	TTATGCAA	3060394	3060546	152 (Pseudogene)
IS3	IS150	ISErh1	1280	ATT/ACC/ATA/CCAG/CGT	323817	324967	1151
	IS150	ISBsu1	1289	TC/GGG/ATA/GAA/CA/-	3195743	3197030	1287
					1207879	1209166	1287
					2873980	2875267	1287
					323722	325008	1286
					1875287	1876573	1286
					2585089	2586375	1286
					962975	964260	1285
					3447123	3448411	1288

ISs determined by after performing a BLASTX analysis in the ISfinder database. All ISs display inverted repeats IR. *ISs displaying two ORFs associated with transposases and hypothetical proteins. ‡IS showing a transposase and a putative acetyltransferase GCN5 in the second ORF. DR: Directed repeats.

Table S18. Genes for putative virulence factors

Predicted virulence-related gene	Product	Identity	Coverage	E-value	Locus tag EA-CB0015 (NZ_CP048852.1)	Locus tag 168 (NC_000964.3)
hlyIII	Hemolysin III	58 %	92 %	6e-62	G4P54_RS11475	BSU_21790
pgsA	Polyglutamic acid capsule	51 %	82 %	8e-108	G4P54_RS18490	BSU_35880
pgsB	Polyglutamic acid capsule	67 %	83%	6e-178	G4P54_RS18500	BSU_35900
pgsC	Polyglutamic acid capsule	71%	99%	2e-59	G4P54_RS18495	BSU_35890
pgsD/ggt	Polyglutamic acid capsule	32 %	89 %	2e-66	G4P54_RS10585	BSU_18410
dhbA	2,3-dihydroxybenzoate-2,3-dehydrogenase (Bacillibactin)	90%	100%	0.0	G4P54_RS16360	BSU_32000
dhbB	Isochorismatase	92%	100%	0.0	G4P54_RS16345	BSU_31970
dhbC	isochorismate synthase	88%	100%	0.0	G4P54_RS16355	BSU_31990
dhbE	2,3-dihydroxybenzoate adenylase	91%	100%	0.0	G4P54_RS16350	BSU_31980
dhbF	non-ribosomal peptide synthetase (Bacillibactin)	89%	100%	0.0	G4P54_RS16340	BSU_31960

Prediction of virulence factors according to the virulence factor database (VFDB) using query genome (*B. tequilensis* NZ_CP048852.1) and VFDB full dataset - all genes related to known and predicted VFs. Locus tag obtained from Genbank database

Table S19. Predicted antimicrobial resistance genes in *B. tequilensis* EA-CB0015

Resistance gene	Identity	Coverage (%)	Position in ref.	Position in query (EA-CB0015)	Locus tag in query (NZ_CP048852.1)	Phenotype	PMID	Accession no. Reference gene
aadK	90.4 %	98.2	1..840	2567437..2568276	G4P54_RS13775	Streptomycin	2550327	M26879
mph(K)	89.7 %	98.0	13..916	259735..260638	G4P54_RS01455	Spiramycin, telithromycin	29317655	NC_000964
tet(L)	87.0 %	99.9	1..1377	3974470..3975846	G4P54_RS01990	Doxycycline, tetracycline	2844261	X08034

ResFinder-4.0 Server results showing the predicted antimicrobial resistance phenotype of *B. tequilensis* EA-CB0015. Phenotype refers to the class of antimicrobial compound the bacterial genome is predicted to be resistant to according to a %ID threshold of 60% and a selected minimum length of 60%.

Table S20. TA systems in *B. subtilis* 168 and their presence in *B. tequilensis* EA-CB0015

**Type 1 TA systems**						Blastn		
**TA system**	**Coordinate**	**Gene name**	**Function**	**Locus tag 168 (NC_000964.3)**	**Locus tag EA-CB0015 (NZ_CP048852.1)**	**Identity %****	**Query cover**	**E value**
txpA/RatA		*txpA*	toxic peptide, eliminates defective cells from developing biofilms	BSU_26050	Absent			
		ratA	control of txpA toxin expression/antitoxin RNA	BSU_misc_RNA_81	Absent			
bsrG/SR4	**1719231-1719460**	***SR4***	**protection of the cell**	**new_2273724_2273988_c**		**74.04%**	**95%**	**6E-30**
	**1719486-1719602**	***bsrG***	**stability of prophage SPß**	**BSU_21546**	**G4P54_RS09095**	**97.44%**	**100%**	**2,0E-52**
bsrE/SR5	**1719496-1719475**	***bsrE***	**maintenance of the prophage-like element P6**	**BSU_18978**		**100%**	**23%**	**2E-04**
	**1719338-1719752**	***SR5***	**control of BsrE toxin expression**	**BSU_misc_RNA_74**		**74.22%**	**98%**	**5E-71**
yonT-yoyJ/SR6	**1775142-1775318**	***yonT***	**unknown**	**BSU_21000**	**G4P54_RS09375**	**99.44%**	**100%**	**8,0E-89**
	**1775337-1775588**	***yoyJ***	**unknown**	**BSU_20999**	**G4P54_RS09380**	**91.67%**	**100%**	**5E-97**
	**1775253-1775356**	***SR6***				**100%**	**100%**	**7,0E-49**
bsrH/as-bsrH		*bsrH*		BSU_26055	Absent			
		as-bsrH	control fo bsrH expression	BSU_misc_RNA_92	Absent			
**Type 2 TA systems**								
NdoA (YdcE, MazF)	**496103-496453**	**ndoA**	**unknown**	**BSU_04660**	**G4P54_RS02550**	**100.00%**	**100%**	**0**
	**495817-496098**	**ndoAI**	**antitoxin, antagonist for EndoA**	**BSU_04650**	**G4P54_RS02545**	**99.29%**	**100%**	**3E-145**
SpoIISA (YkaC)/SpoIISB/ SpoIISC	**1265825-1266571 1265655 1265825**	**spoIISA**	**Toxin/lethal when synthesized during vegetative growth in the absence of SpoIISB**	**BSU_12830**	**G4P54_RS06785**	**93.17%**	**100%**	**0**
		**spoIISB**	**Antitoxin/disruption blocks sporulation after septum formation**	**BSU_12820**	**G4P54_RS06780**	**94.74%**	**100%**	**4,0E-72**
		**spoIISC**	**anti-toxin to SpoIISA**		**G4P54_RS06775**			

Yeef/YezG	**715016-717025**	**yeeF**	**Polymorphic toxin, non-specific metal-dependent Dnase/competetion with other bacteria**	**BSU_06812**	**G4P54_RS03725**	**94.15%**	**96%**	**0**
	**713492-713947**	**yezG**	**Antitoxin/inhibition of the cytotoxic activity of YeeF**	**BSU_06811**	**G4P54_RS03710**	**96.05%**	**100%**	**0**
yobK/yobL		yobK	inhibition of the cytotoxic activity of YobL	BSU_18990	Absent			
	1714949-1716859	yobL	Toxin/unknown	BSU_19000	G4P54_RS09070	91.84%	82%	0
yokI/yokJ		yokI	putative toxin	BSU_21580	G4P54_RS09070	91.39%	68%	0
		yokJ	putative antitoxin/inhibition of the cytotoxic activity of YokI	BSU_21570	Absent			
yqcF/ywqG		yqcF	antitoxin/inhibition of the cytotoxic activity of YqcG	BSU_25870	Absent			
		yqcG	toxin/eliminates defective cells from developing biofilms	BSU_25860	Absent			
ywqJ/ywqK	3549018-3550186	ywqJ	putative toxin/unknown	BSU_36190	G4P54_RS18655	94.27%	64%	0
		ywqK	BSU_36180/unknown	BSU_36180	Absent			
yxiD/yxxD	3857250-3858484	yxiD	Toxin/unknown	BSU_39300	G4P54_RS20250	92.15%	72%	0
		yxxD	Antitoxin/inhibition of the cytotoxic activity of YxiD	BSU_39290	Absent			

In bold: complete TA systems

Supplementary Figures

Figure S1. Cumulative GC skew for the genome of *B. tequilensis* EA-CB0015

The x-axis represents the chromosome position, and the y-axis represents the G/C values. The symmetric GC skew for this genome predicts the loci of the origin of replication (ORI) to start at position 1 of the chromosome and the terminus of replication (TER) to be located at ca. 1.9 Mb. SkewDB (https://skewdb.org) was used to obtain the GC skew of this strain.

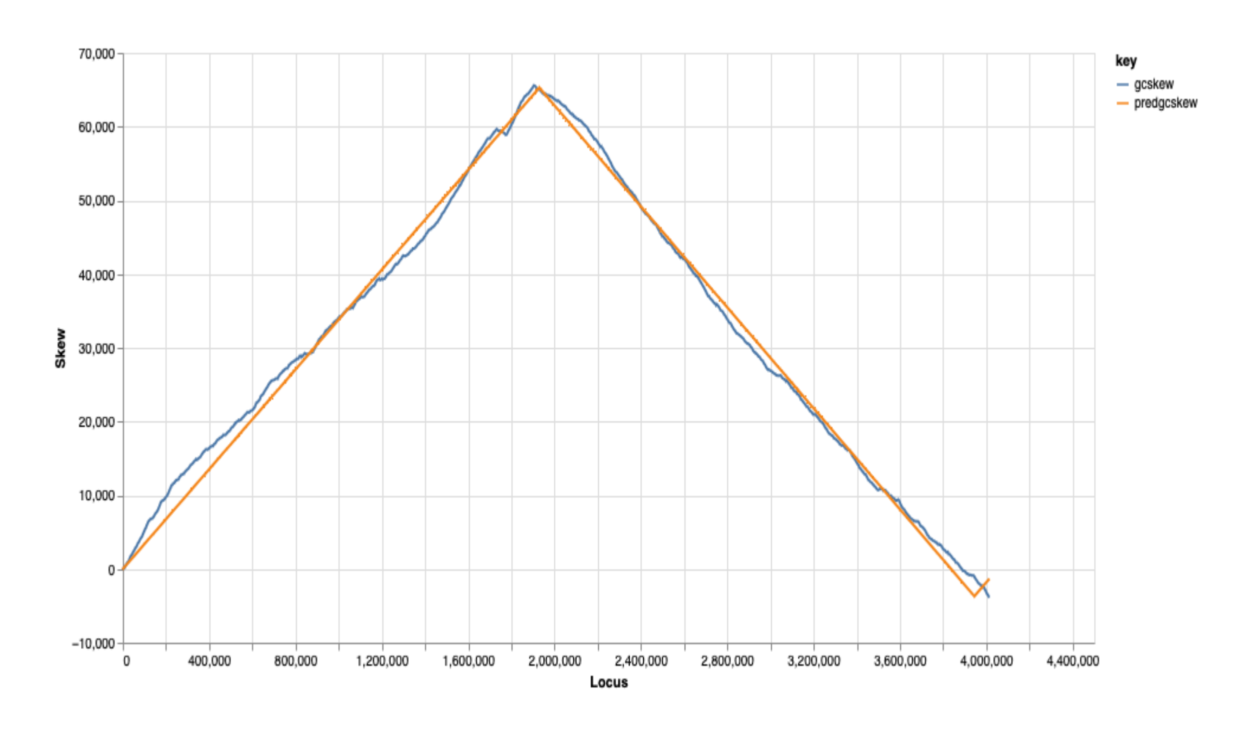

Figure S2. Distribution of clusters of orthologous groups (COGs) for *B. tequilensis* EA-CB0015

Numbers indicated in the horizontal bars indicate the number of families assigned to each COG category

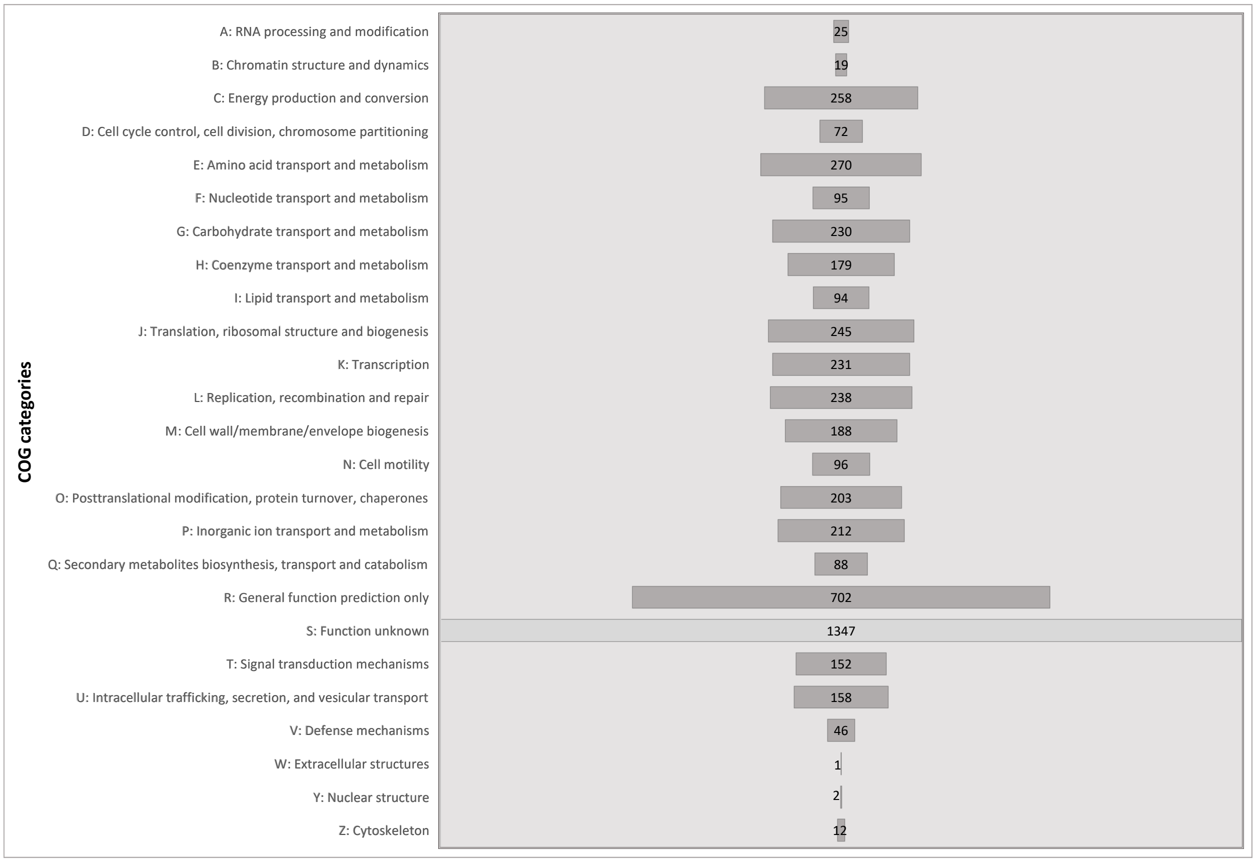

Figure S3. Multicellular lifestyle traits of *B. tequilensis* EA-CB0015.

a) Micrograph showing flagellated cells using Ryu stain. White arrows point at the detected flagella. **b)** Swarming and swimming motility assays performed in 10% LB agar supplemented with 0.7% or 0.3% agar, respectively. **c)** Biofilm formation ability assessed in LBGM media. Micrograph represents the top view of well. The wrinkly formation developed in the liquid air interphase **d)** Endospore formation assessed using Schaeffer-Fulton staining method. Green rounded structures correspond to spores and safranine colored rods represent vegetative cells.

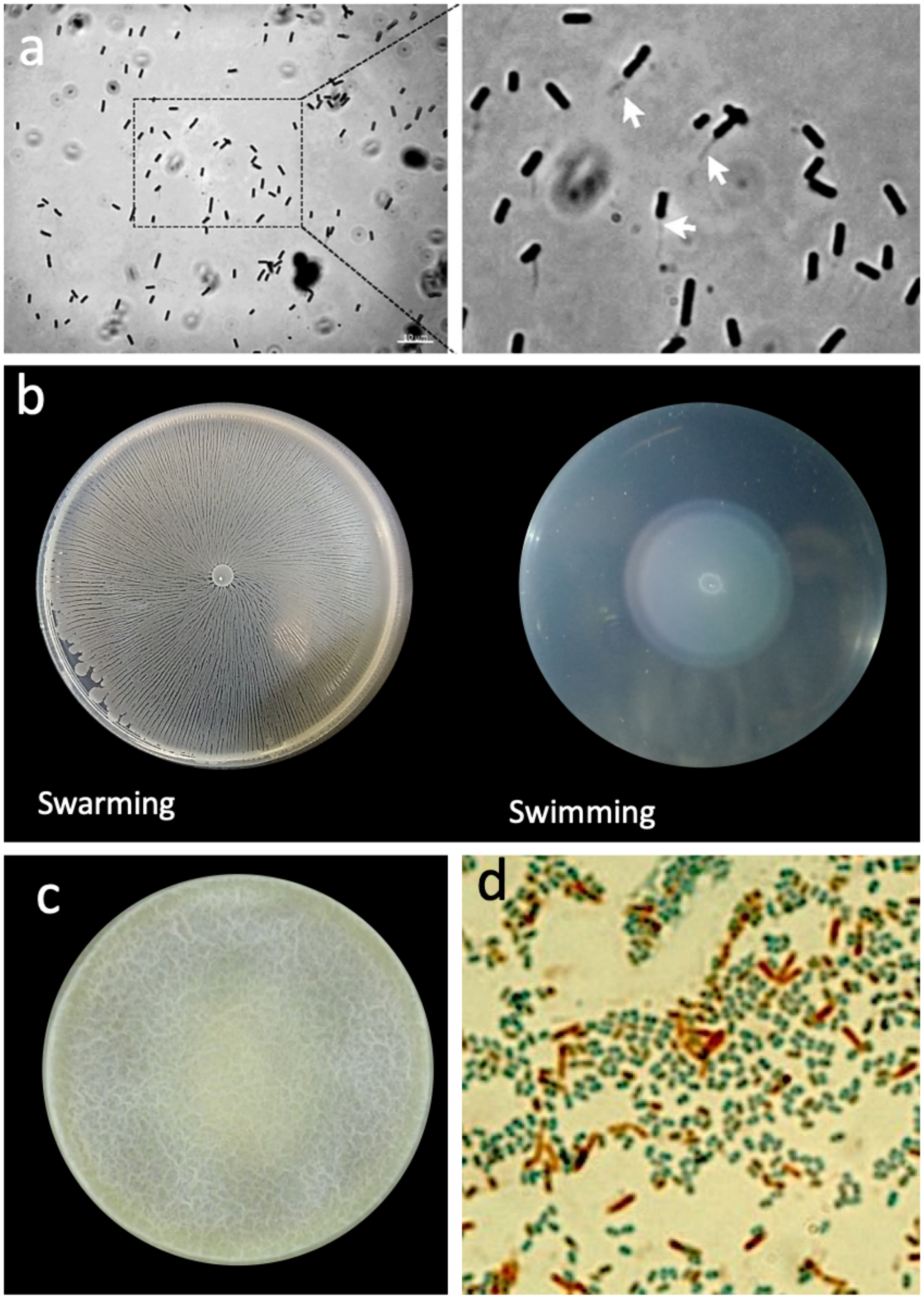

Figure S4. Synteny of natural product BGCs from *B. tequilensis* EA-CB0015 against the reference sequences

Type	Name	Ref. known cluster	Similarity	Comparative Synteny Analysis
NRPs	Fengycin	CP000560.1	100%	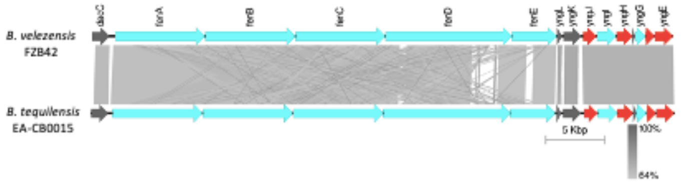
	Iturin	AB050629.1	90%	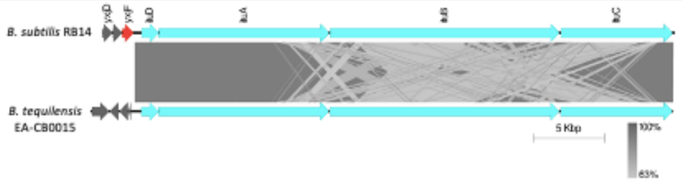
	Surfactin	AJ575642.1	69%	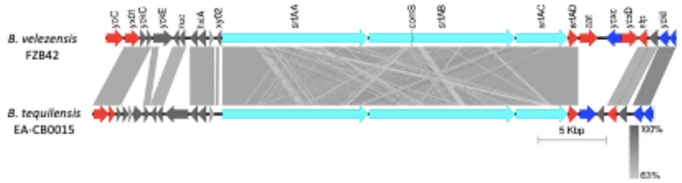
	Bacillibactin	AL009126.3	100%	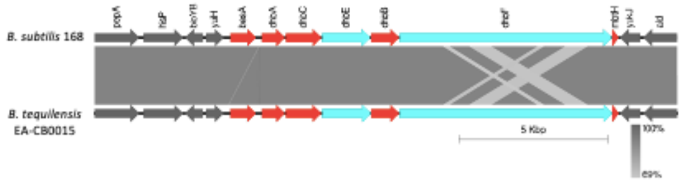
	Bacilysin	CP000560.1	100%	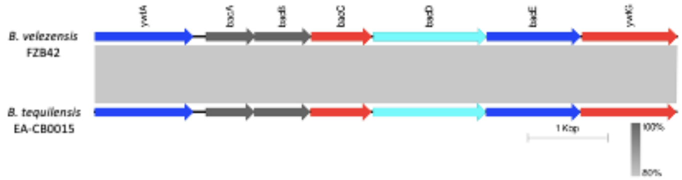
RiPPs	Subtilosin A	AJ430547.1	87%	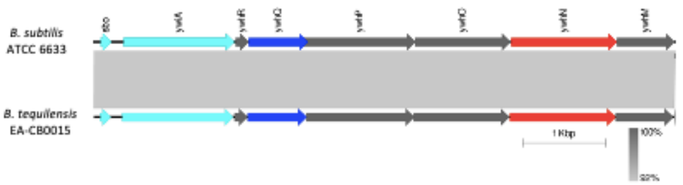
	Sporulation killing factor (SKF)	AL009126.3	100%	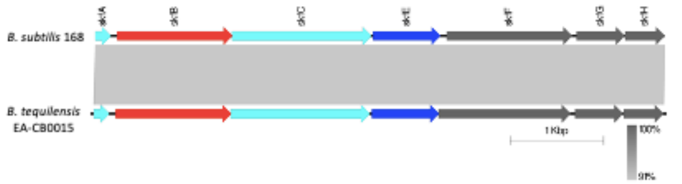

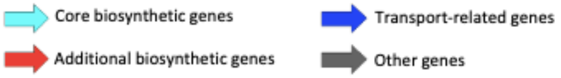

Figure S5. Prophages in the genome of *B. tequilensis* EA-CB0015

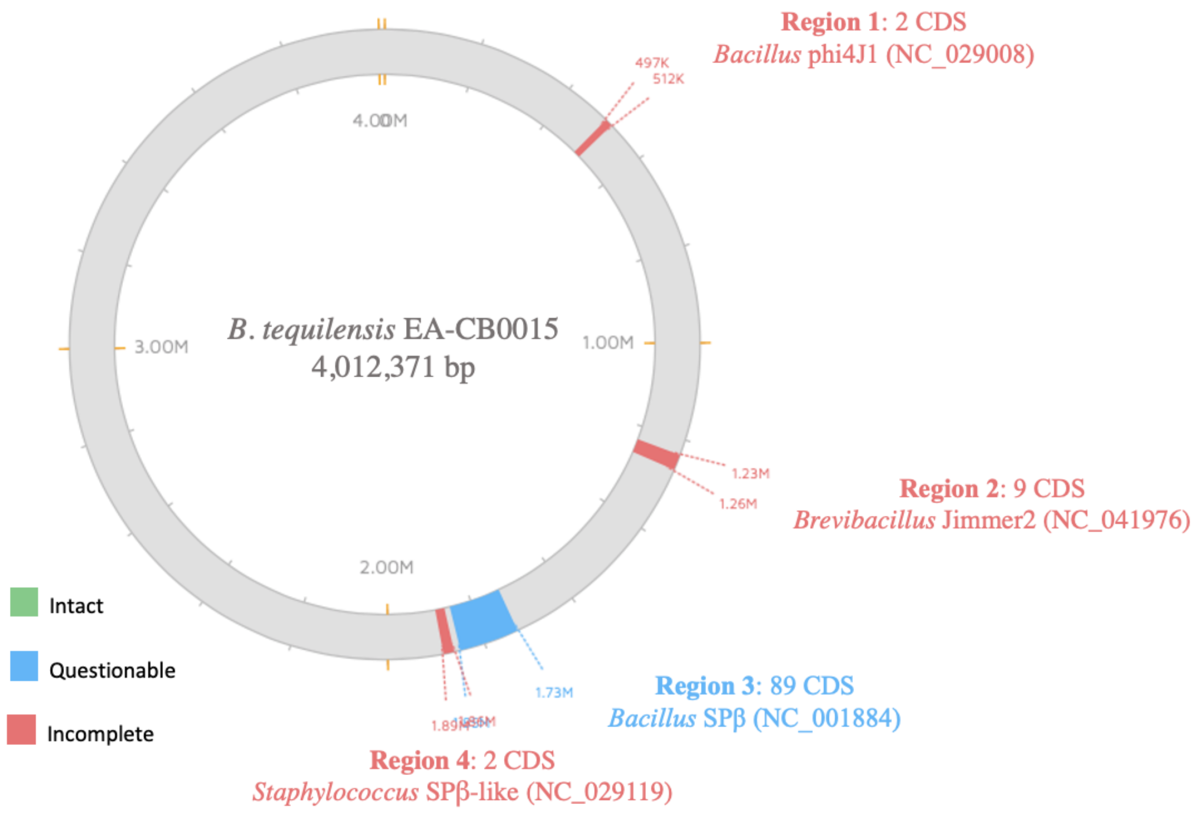

Figure S6. Circular view of the EA-CB0015 genome highlighting genomic islands (GIs) Different prediction methods were used and are illustrated with the different colors. Track 1 in red: shows the integrated results obtained by the different prediction methods and highlights the region that was predicted by at least one method (IslandPath-DIMOB, IslandPath-DIMOB, SIGI-HMM and IslandPick); track 2 in blue: prediction made by IslandPath-DIMOB; track 3 in orange: prediction made by SIGI-HMM; track 4 in green: prediction made by IslandPick; track 5 shows a single pink circle around position 3.6M: represents a homolog of the antimicrobial resistance gene *murA*, related to conferring resistance to fosfomycin.

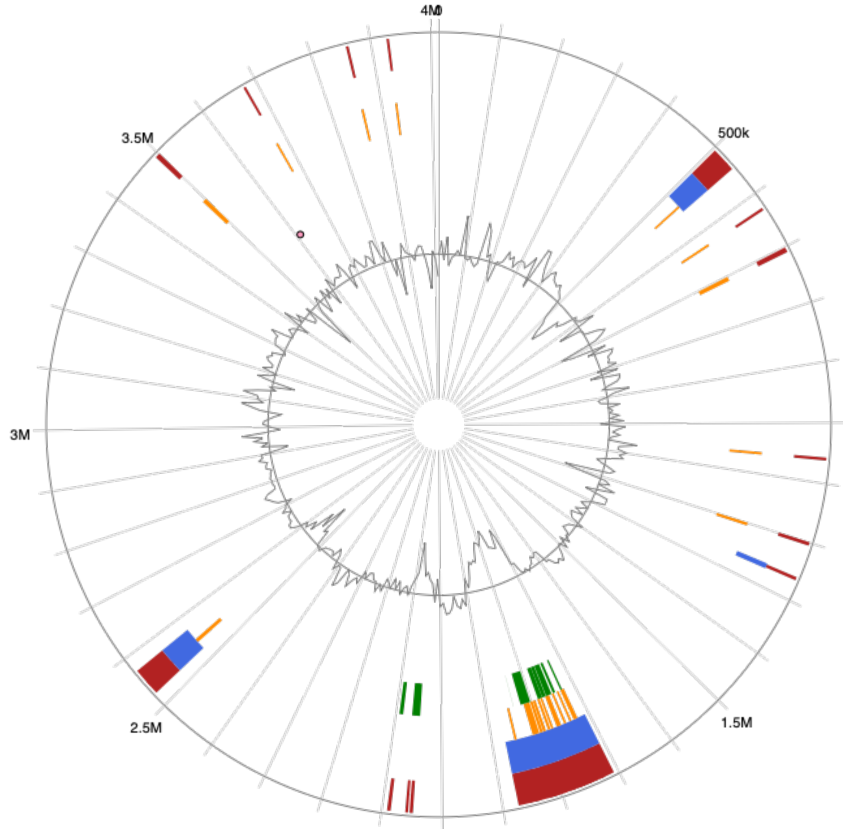

Figure S7. Type I restriction modification system identified from REBASE
The graph indicates the genomic location of the gene cluster containing the genes coding for the subunits M (Methylase), S (specificity DNA site), and R (restriction endonuclease).

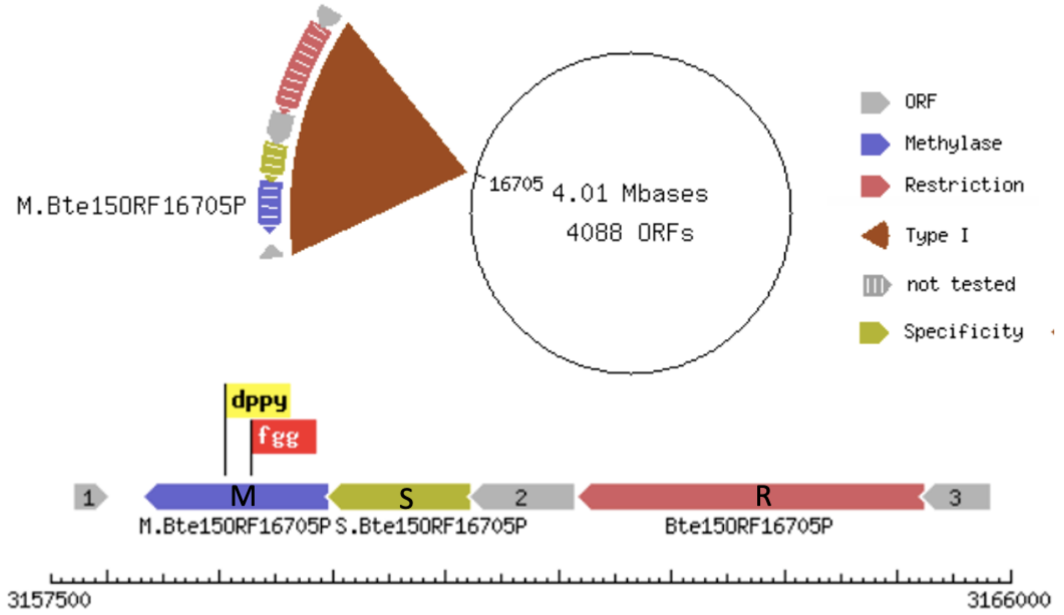

Locus tag	start	end	Function	Name (REBASE)
G4P54_16700	3157723	3157995	Hypothetical protein	1
G4P54_16705	3158343	3159959	Type I restriction-modification system subunit **M**	M.Bte150ORF16705P
G4P54_16710	3159976	3161214	Restriction endonuclease subunit **S**	S.Bte150ORF16705P
G4P54_16715	3161228	3162133	Hypothetical protein	2
G4P54_16720	3162176	3165232	Type I restriction endonuclease subunit **R**	Bte150ORF16705P
G4P54_16725	3165237	3165815	Restriction endonuclease subunit **S**	3

Figure S8. Top 14 results from cluster BLAST of the EA-CB0015 type I RM system
Colors indicate different subunits from the RM system. The cluster score and rank are calculated based on the cluster similarity, that is the number of sequences with BLAST hits and the number of contiguous gene pairs with conserved synteny calibrated with a weight factor (*i* = *0.5*). The genomic coordinates for the cluster hit are retrieved based on Identical Protein Groups (IPG) resources. Identity values for subunit S was showen as the others display values greater than 85 %. Clusters were generated using Cblaster v1.3.0. RM system genes consist primarily of methyltransferase (M subunit; G4P54_16705/G4P54_RS16755), restriction endonuclease (R subunit; G4P54_16720/G4P54_RS16770) and specificity DNA site (S subunit; G4P54_16710/ G4P54_RS16760) genes. Three hypothetical genes (1: G4P54_16700/ G4P54_RS16750; 2: G4P54_16715/ G4P54_16765 and 3: G4P54_16715/ G4P54_RS16775) are predicted with unknown or presumed function.

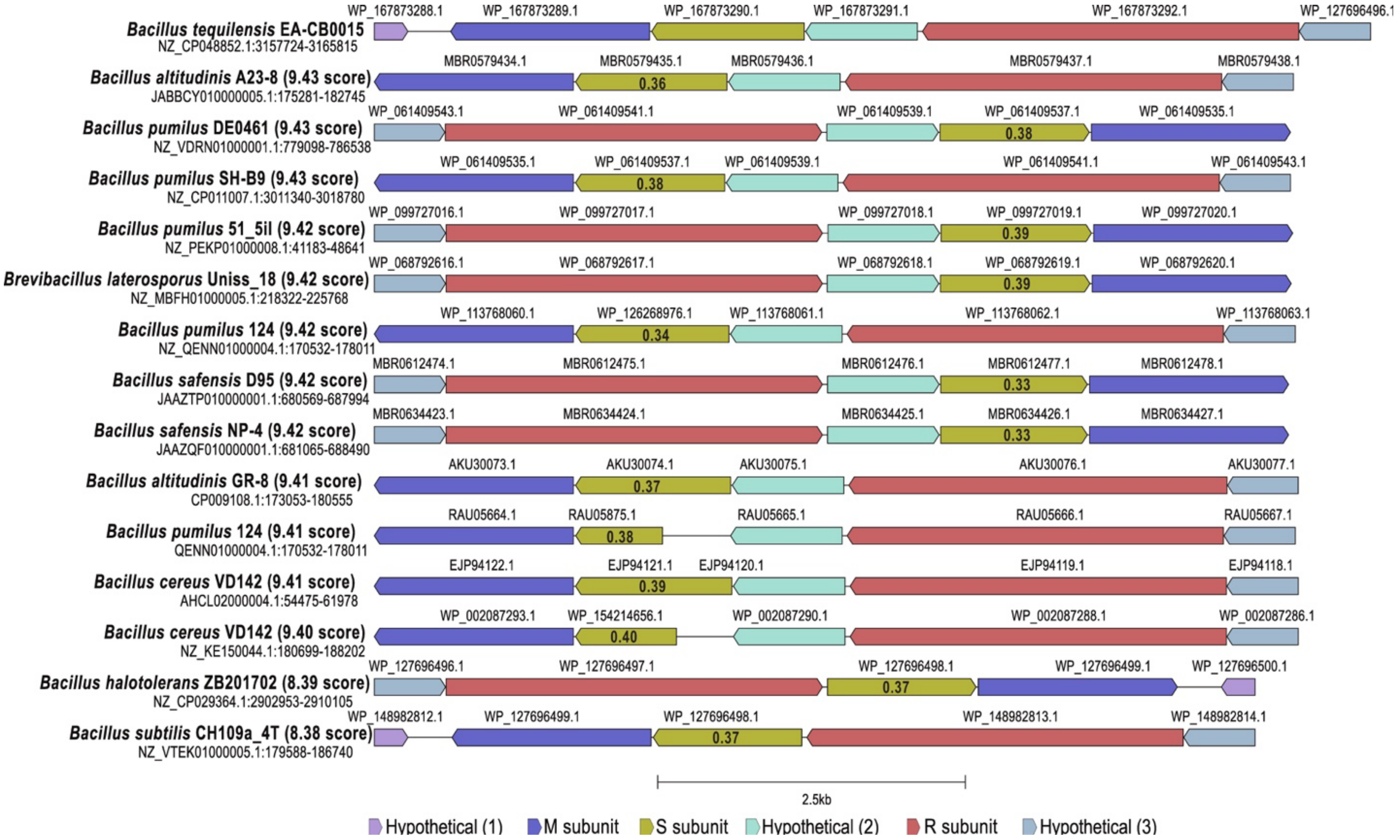

Figure S9. Location of type II toxin/anti-toxin systems identified by TAfinder

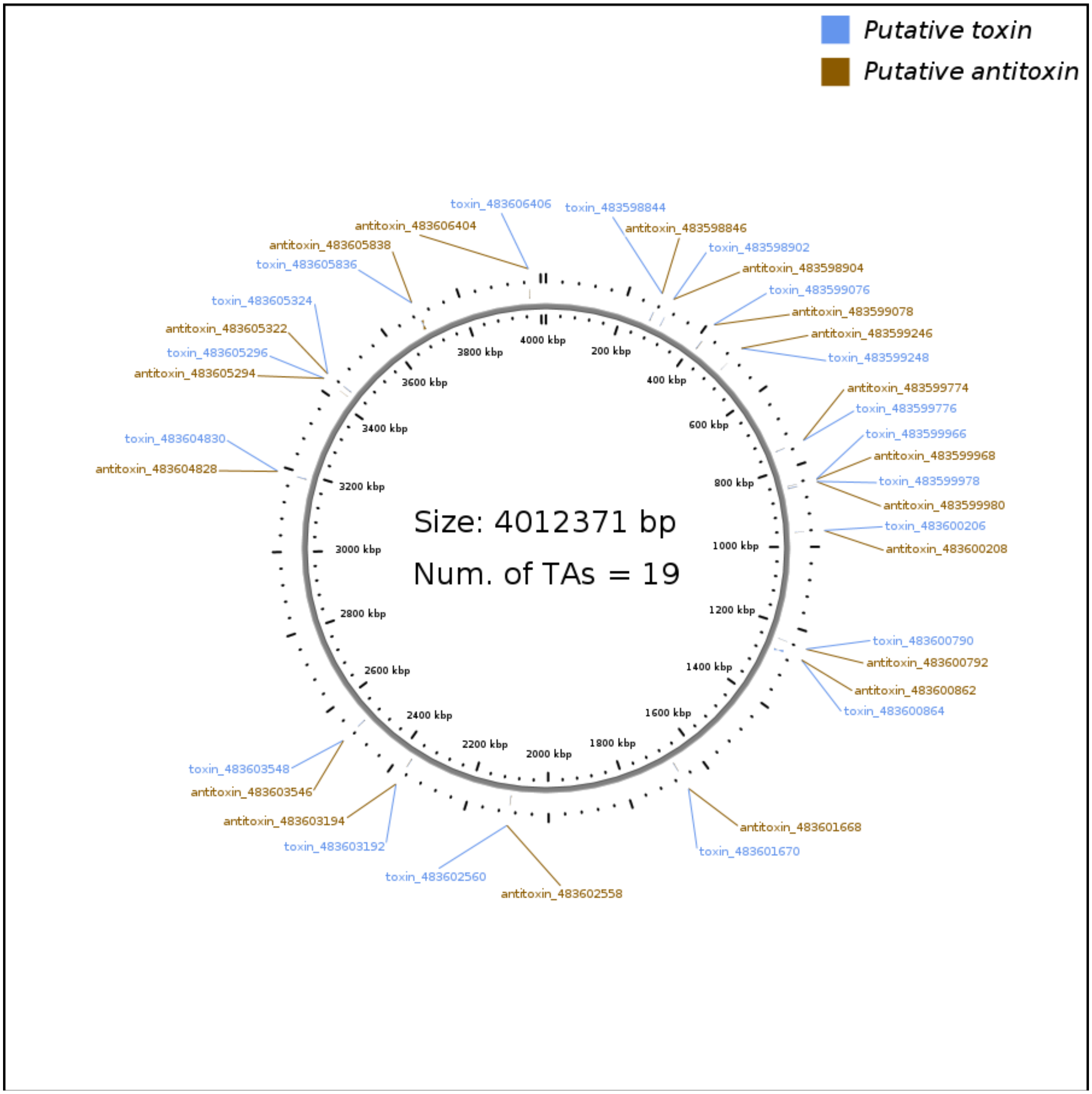

Figure S10. Comparisons of prophage regions between *B. subtilis* and *B. cereus* complex

Total number of predicted prophage regions identified (A). Number of CDSs present in predicted prophage regions (B). Number of prophage regions grouped by level of completeness: incomplete (C), intact (D) and questionable (E). Number of CDSs present in predicted prophage regions grouped by level of completeness (F-H). Analysis are made based on the results obtained using PHASTER. All comparisons were performed using parametric *t-test*; no significant differences were observed.

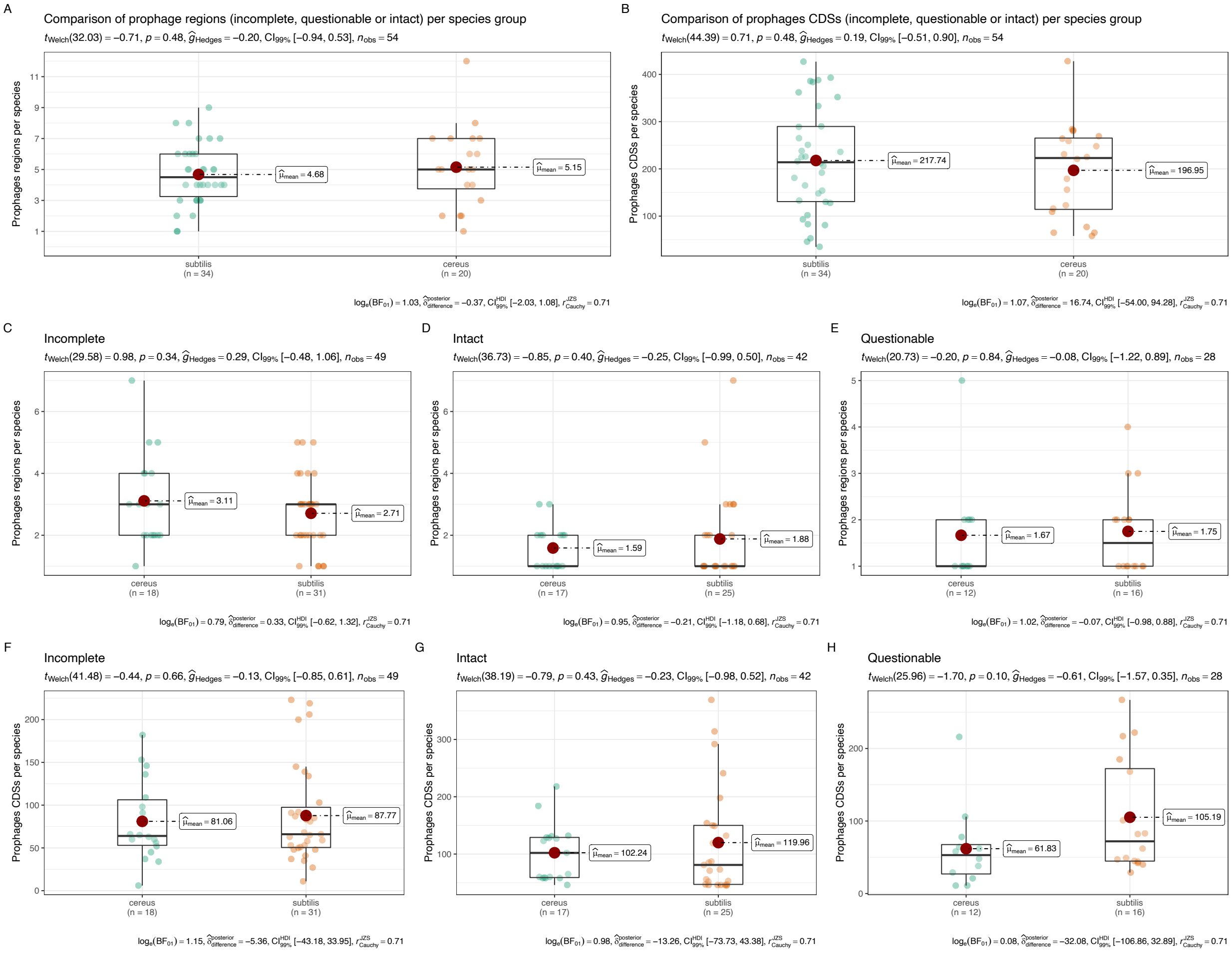

Figure S11. Diversity of intact prophage regions found in the genomes of different *Bacillus* species

Prophage CDSs were identified with PHASTER

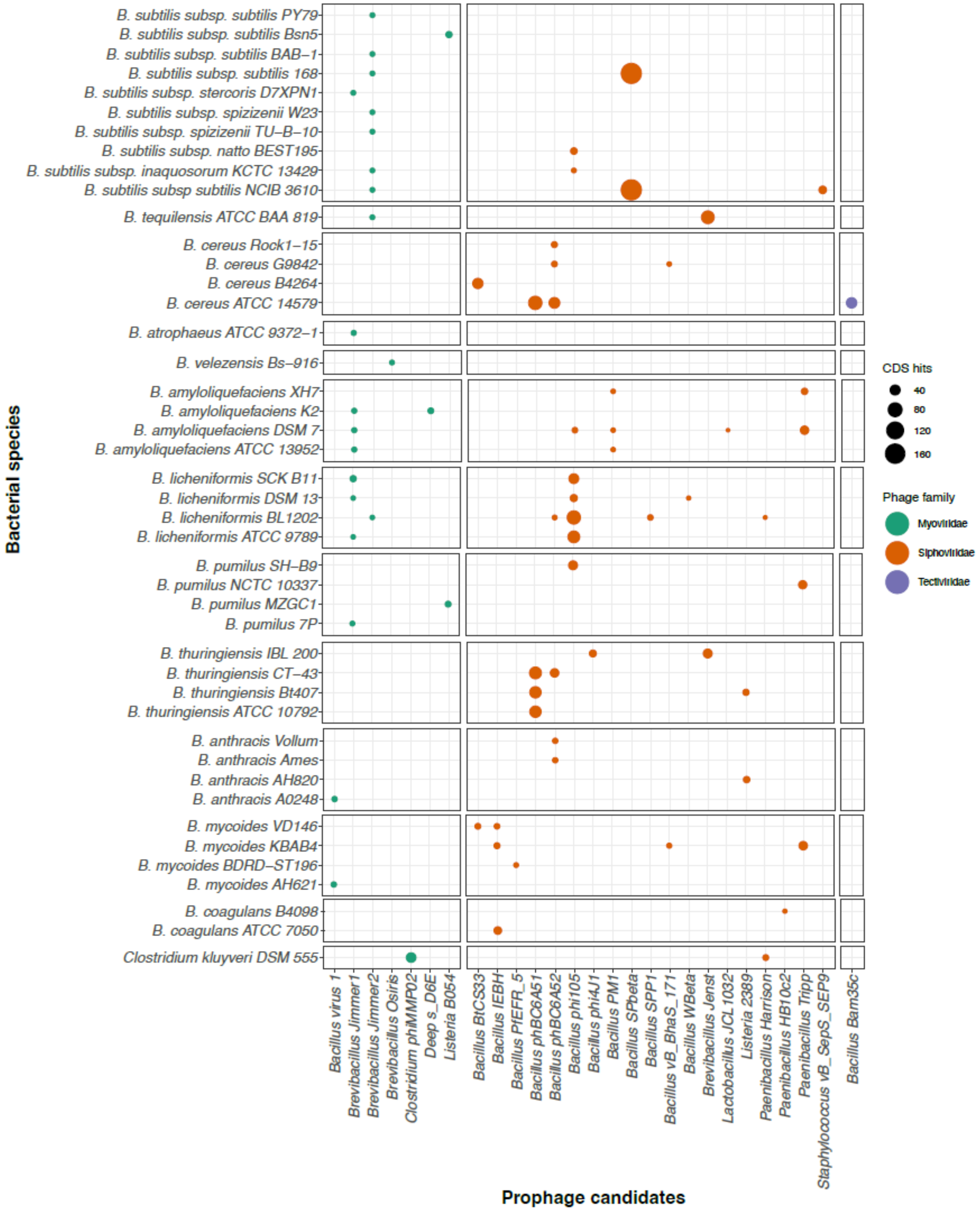

Figures S12. Venn diagram of intact phages reported for *B. subtilis* complex and *B. cereus* complex based on the MLSA

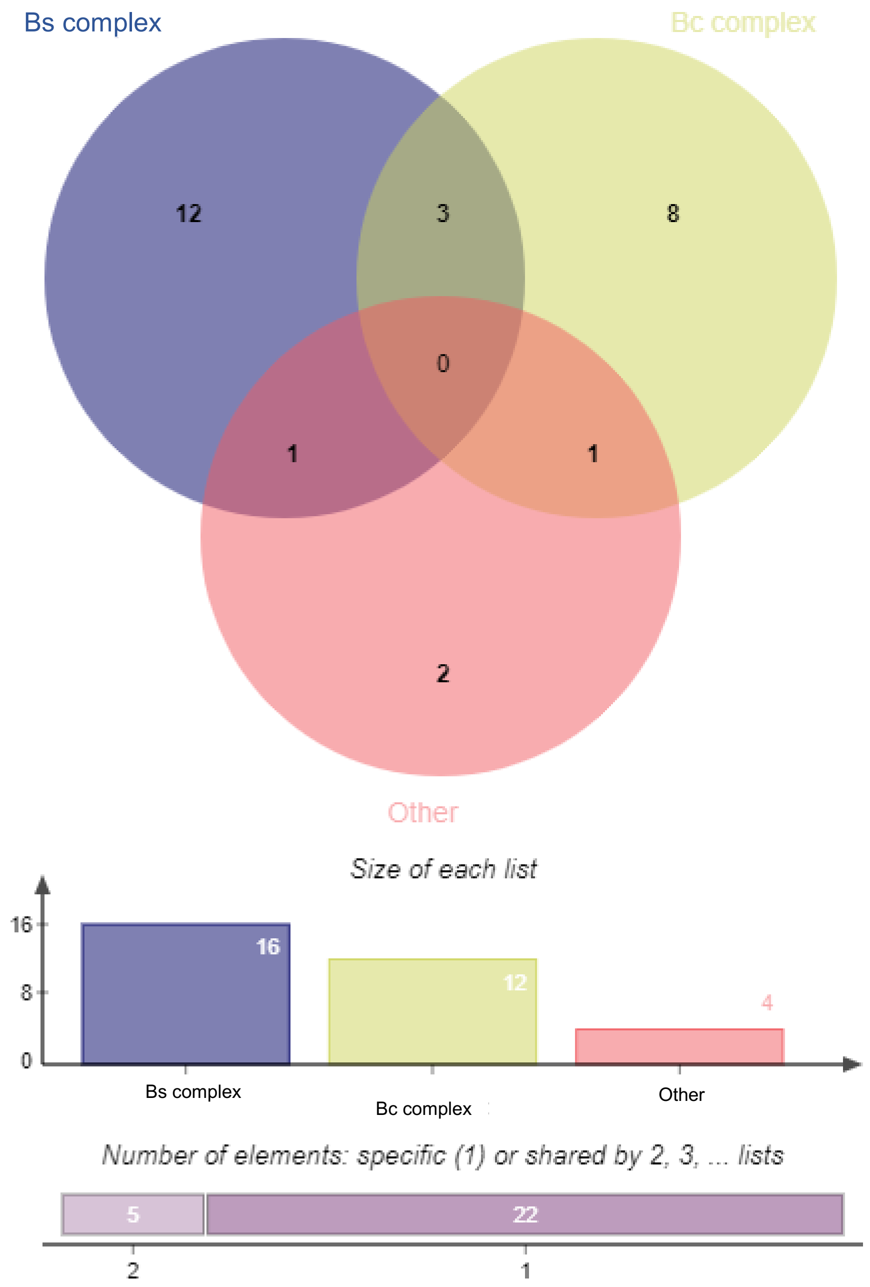

Supplement: Supplementary file 1 [file Data_Sheet_1.PDF]
